# Models for Predicting the Architecture of Different Shoot Types in Apple

**DOI:** 10.3389/fpls.2017.00065

**Published:** 2017-02-01

**Authors:** Emna Baïram, Mickaël Delaire, Christian Le Morvan, Gerhard Buck-Sorlin

**Affiliations:** Unité Mixte de Recherche 1345, Institut de Recherche en Horticulture et Semences (Institut National de la Recherche Agronomique-Agrocampus Ouest-Université d'Angers)Angers, France

**Keywords:** *Malus x domestica* Borkh., apple, leaf surface, shoot architecture, allometry, modeling, apple branch

## Abstract

In apple, the first-order branch of a tree has a characteristic architecture constituting three shoot types: bourses (rosettes), bourse shoots, and vegetative shoots. Its overall architecture as well as that of each shoot thus determines the distribution of sources (leaves) and sinks (fruits) and could have an influence on the amount of sugar allocated to fruits. Knowledge of architecture, in particular the position and area of leaves helps to quantify source strength. In order to reconstruct this initial architecture, rules equipped with allometric relations could be used: these allow predicting model parameters that are difficult to measure from simple traits that can be determined easily, non-destructively and directly in the orchard. Once such allometric relations are established they can be used routinely to recreate initial structures. Models based on allometric relations have been established in this study in order to predict the leaf areas of the three different shoot types of three apple cultivars with different branch architectures: “Fuji,” “Ariane,” and “Rome Beauty.” The allometric relations derived from experimental data allowed us to model the total shoot leaf area as well as the individual leaf area for each leaf rank, for each shoot type and each genotype. This was achieved using two easily measurable input variables: total leaf number per shoot and the length of the biggest leaf on the shoot. The models were tested using a different data set, and they were able to accurately predict leaf area of all shoot types and genotypes. Additional focus on internode lengths on spurs contributed to refine the models.

## Introduction

The study of plant architecture is a discipline that attempts to understand and explain plant form and structure and the processes underlying its formation (Barthélémy, 1991). Vascular plants have developed different architectures as part of their genetic blueprint and in response to a changing environment (Sussex and Kerk, 2001). The size, shape and spatial orientation of plant organs are, therefore, not pure coincidence but the result of a morphogenetic program which is carried out by a whole range of physiological processes. Therefore, “reading” the architecture could be a starting point for a better understanding of this underlying program. Of these architectural traits, leaf area has a particular impact on fruit quality as it is directly involved in several physiological processes such as light interception and photosynthesis (Björkman and Demmig-Adams, 1995; Štampar et al., 1999).

Studies on the influence of tree architecture on physiological functioning can be conducted in several ways: Two suitable tools are ecophysiological experimentation and functional-structural plant modeling (FSPM) (Vos et al., 2010; Buck-Sorlin, 2013): Ecophysiological experiments aim at changing the microenvironment of a selected plant and its organs and even at pushing it to an extreme limit, in order to obtain knowledge about the growth and developmental potential in a certain parameter space. FSPM aims at the integration of the dynamics of known physiological processes with information about the topology and geometry of organs (plant architecture) using mainly rule-based mathematical modeling (Buck-Sorlin, 2013).

In order to represent initial architecture there are several methods at hand and it is worthwhile to invest some time in developing a work flow to obtain “good” plant architecture with reduced effort. Casella and Sinoquet (2003) name several approaches: (1) describe architecture as a collection of individual 3-D geometric shapes; (2) model 3D architecture of a population of plants using stochastic, fractal or Lindenmayer-system (Lindenmayer, 1968a,b) methods or (3) describe architecture using a 3D digitizing method. All these methods have their advantages and disadvantages (for a review see Prusinkiewicz, 1998): The first method is suitable for the representation of the context of a detailed tree model, but too coarse for the modeling of leaf photosynthesis. The second one, despite being relatively quickly put into place, can still turn out to be too inaccurate for the description of leaf-scale photosynthesis since due to the stochastic method of architecture construction the reproducibility of a given real architecture is difficult. Apart from that, this method requires extensive calibration with measured data sets. The third method, though the most accurate one, is unsuitable for logistic reasons in the orchard: in the absence of a socket an electricity generator needs to be used, and there might be interference with the steel wires used for fixing the drip irrigation system, quite apart from the fact that digitizing is a tedious task and the structure to be digitized is often too complex to be acquired in 1 day.

Yet another, alternative approach is to use allometric relations between traits at the organ and intermediate (shoot, branch) scales: The principle is to obtain faithful models for the prediction of traits that are difficult to measure or that involve destruction of the organ, e.g., leaf area, by traits that are more readily measured (e.g., leaf blade length) or easily and non-destructively scored in the orchard (leaf number, rank, order). Once such allometric relations are established they can be used routinely to recreate initial structures. As with all indirect measures they need to be well tested as they bear the risk of cumulative error.

**Figure 1:**
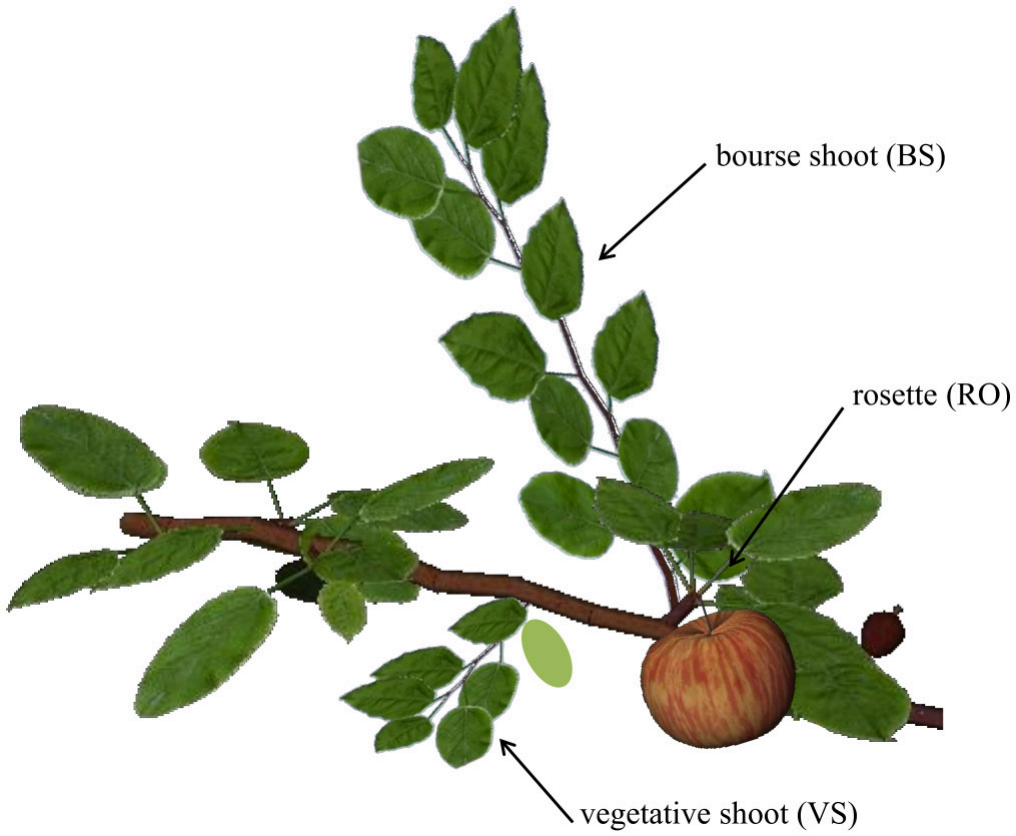
**Representation of the three shoot types on the first-order branch of apple**. RO, rosette; BS, bourse shoot; and VS, vegetative shoot. Figure produced using the GroIMP platform (Hemmerling et al., 2008).

In apple two types of buds are distinguished: the mixed and the vegetative bud. The mixed bud, independent of its position on the shoot (apical or lateral), contains primordia of vegetative and reproductive organs and will develop into a spur. Thus the spur consists of a short shoot called “bourse” on which the primordia of preformed leaves will extend, as well as one or two sylleptic shoots called “bourse shoots” and the inflorescence (Fanwoua et al., 2014). The vegetative bud develops into a vegetative shoot. In temperate species, short axes are composed of preformed organs only whereas long axes are composed of both preformed and neoformed organs successively (Costes et al., 2014); therefore, branch architecture is essentially determined by the developmental fate of these two bud types (Figure 1).

In this study we decided to concentrate on the prediction of leaf area: of all the traits contributing to branch architecture (leaf area, leaf blade and petiole orientation in space), it is the one that is most easily determined (as will be shown below) and yet very influential for light interception (Falster and Westoby, 2003). It was shown for apple that leaf area was the second most influential trait for light interception, after internode length (Da Silva et al., 2014). The distribution of leaves on the shoot and their size distribution clearly have an influence on light interception and leaf photosynthesis at the branch scale (Massonnet et al., 2008).

Any good model should be minimal in terms of number of input variables and the time invested in measurements but also efficient in simulating the output variable of interest. Therefore, the main aim of this study was to find models predicting the area of leaves of the three main shoot types of apple: bourse shoot (BS), rosette (RO) and vegetative shoot (VS), and this as a function of other traits on the same shoot that were either easy to score (total leaf number per shoot) or relatively quickly measured (length of the longest leaf of a given shoot). A secondary aim was to model the position of leaves on the shoot (length of the internodes) as a function of simple measured traits (length of the shoot). Such leaf area distributions can then serve to reconstruct the architecture of an apple branch used as an input for a functional-structural plant model of the first-order branch of apple, with emphasis on sugar transport (Bairam et al., unpubl.).

## Materials and methods

### Plant material and experiments

All experiments were performed on apple trees planted in 2008 in an experimental orchard at the INRA experimental unit in Beaucouzé, France. The cultivars selected for this experiment were “Ariane,” “Fuji,” and “Rome Beauty” (in the following abbreviated as AR, FU, and RB, respectively). The main purpose of this study was to develop an allometric model for the prediction of the distribution of individual leaf areas along a certain shoot type, as well as of the total leaf area for a given shoot, namely the bearing spurs and vegetative shoots as they are the most cumbersome to be measured in the orchard. The models presented here were developed to take into account the impact of the genotype (G, with values AR, FU, and RB, see above) and the type of the shoot (*j*, which has the value “BS” for the bourse shoot, “RO” for the rosette and “VS” for the vegetative shoot). Shoots of each type were collected, and the leaves were scanned using a flatbed scanner (HP Scanjet G4010) with a resolution of 150 dpi and saved in portable network graphics (png) format. Leaf blade area, length and width were measured using ImageJ 1.48v software, assuming that leaf shape is elliptical (Freeman and Bolas, 1956). Total leaf area on each shoot was calculated as the sum of its individual leaves.

The models predicting individual/total leaf area use allometric relations between easily recorded input variables such as the length of the ellipse of the biggest leaf on the shoot (*L*_*max*_), the number of leaves (*nl*) and the acropetal leaf rank (*R*). These models were built using different parameters and each of them was estimated using regression models. For rosettes and bourse shoots, enough data was available to be split into training and testing sets: the first data set was used for the estimation of the parameters and the second one for the testing of the model (Snee, 1977; Montgomery et al., 2015). Therefore, models were parameterized, calibrated and tested. For vegetative shoots, a simple model was built as there was not enough data for testing it.

### Modeling the leaf area of bourse shoots and rosettes

Data used in this study consisted of a first data set from an experiment conducted in 2014 and a second, complementary data set collected in 2015 (Bairam et al., unpublished). The variables describing architectural traits of bourse shoots and rosettes [shoot length, number of leaves, leaf individual surface, leaf length, leaf width, and leaf rank (the latter only for bourse shoots)] extracted from the two sets were used for modeling total shoot leaf area and individual leaf area distribution along the stem. In the 2014 experiment, spurs of AR and RB were collected at eight different developmental stages from full bloom to harvest, and spurs of FU were collected at full bloom, 2 weeks after full bloom and at harvest. The experiment conducted in 2014 aimed at studying the impact of removing bourse shoot leaves, rosette leaves or both of them at full bloom and 2 weeks after full bloom, on fruit quality, bourse shoot and rosette (bourse) morphological traits. To develop an allometric model for the prediction of final leaf area, we needed a sufficiently large data set. However, the data set available in this study (2014 experiment, Bairam et al., unpublished) already included three defoliation treatments. In order to enlarge the database for the parameterization of our model beyond the control data, we checked whether the defoliation treatments had a significant impact on the following variables used in the model: total leaf area and number of leaves of bourse shoot and rosette, respectively. The statistical tests were carried out separately, on each genotype for each sampling date and each type of shoot. Results (ANOVA, Kruskal-Wallis) showed that there was no significant influence of the defoliation treatments (Bairam et al., unpublished). This allowed us to pool the data available for these variables, involving all defoliation treatments, and to use them in the model. 174, 49, and 134 rosettes of AR, FU and RB, respectively, as well as 336, 80, and 280 bourse shoots of AR, FU and RB, respectively, were retained from the 2014 experiment. Afterwards, for AR and RB, tests (ANOVA, Kruskal-Wallis) were conducted on 2014 data in order to establish the phenological stage of development expressed in growing degree days (GDD) since FB from which onwards each type of shoot for each genotype was fully developed in terms of number of leaves and total leaf area. This developmental time was first measured using growing degree hours [GDH, base temperature *T*_*b*_ = 7°C (Anderson and Richardson, 1982)] calculated using hourly air temperatures (°C) obtained from the weather station of Beaucouzé (47° 28′ N, 0° 37′ W, 50 m a.s.l.) and accessed from the INRA Climatik platform, https://intranet.inra.fr/climatik_v2/:

(1) $$\begin{array}{l} GDH_{i}\;=\;\sum_{h=1}^{24}\left(HT_{h}\;-\;T_{b}\right) \end{array}$$

Here, *HT*_*h*_ is replaced by *T*_*b*_ if *HT*_*h*_ < *T*_*b*_; *HT*_*h*_ is the hourly air temperature at hour *h*; and *GDH*_*i*_ are the growing degree hours on day *i*. Cumulated GDD (*GDD*_*cum*_) for each sampling day were calculated using Equation 2. The starting point of *GDD*_*cum*_ is full bloom (FB) which, in 2014, occurred for AR on April 10th, for FU on April 14th and for RB on April 22nd while in 2015, full bloom occurred for FU on April 20th.

(2) $$\begin{array}{l} GDD_{cum}\;=\;\sum_{i\;=\;FB}^{D}\frac{GDH_{i}}{24} \end{array}$$

where *FB* is day of full bloom and *D* is the number of days since *FB*.

**Figure 2:**
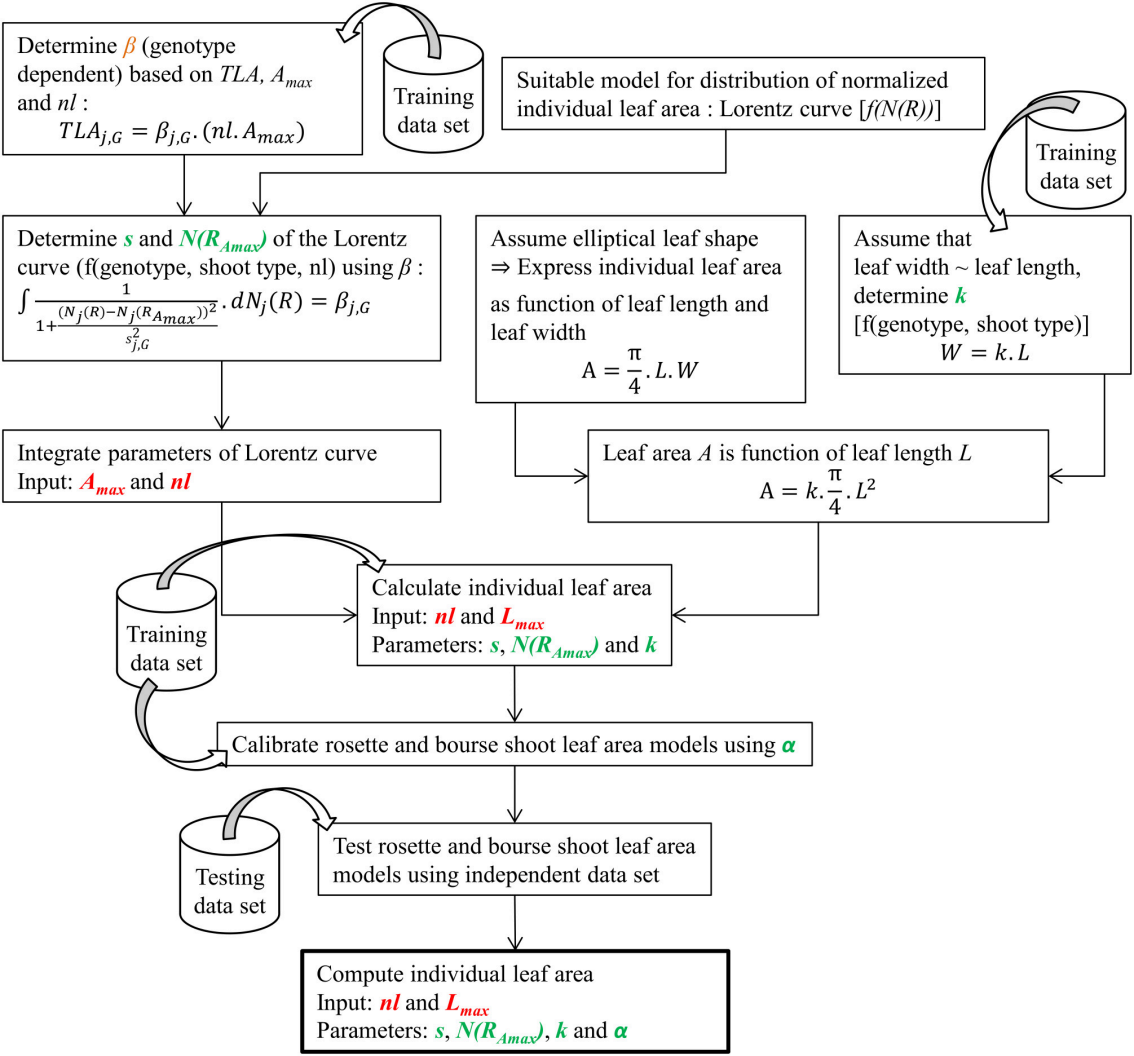
**Diagram summarizing the steps involved in modeling bourse shoot and rosette individual leaf area (for further explanations see text and equations)**.

During the second experiment conducted in 2015, the same variables as in 2014 were recorded plus the ranks of rosette leaves; however, based on the results of the 2014 experiment no defoliation treatments were applied and only spurs of FU at different phenological stages (FB, FB+2, FB+4, FB+6, FB+9 weeks) were considered. For FU, ANOVA and Kruskal-Wallis analysis were carried out on 2015 and 2014 data to compare total leaf area and number of leaves (rosettes and bourse shoots) as a function of developmental stages (expressed in GDD) in order to establish the sampling stages at which the spur shoots were fully developed. Therefore, fully developed bourse shoots and rosette shoots, respectively, were pooled for each genotype. All shoots of the same type and the same genotype considered to be fully developed in terms of total leaf area and numbers of leaves were used in this study for building the allometric models. Total leaf areas and numbers of leaves of bourse shoots of AR, FU, and RB, were fully developed at 345, 201, and 275 GDD, respectively. Groups of bourse shoots of each genotype were selected from these physiological ages onwards, until harvest. Rosette shoots were assumed to be fully developed at full bloom as the mean total leaf areas for the three genotypes were the highest at the earliest sampled spurs. However, even if a significant difference was found between groups of rosettes sampled at different phenological stages for a same genotype, still the means are not correlated with phenological sampling dates and the only group really apart in terms of rosette total leaf area and number of leaves for the three genotypes, respectively, was the one collected at harvest (Bairam et al., unpublished). Therefore, only rosettes collected before harvest were retained for the rest of the study. Only selected spurs were used for the rest of this study and data selected for each genotype and each shoot type was split randomly into a training set (2/3 of data) and a testing set (the remaining 1/3 of data) for setting up the models. The procedure to build the models for predicting bourse shoot and rosette individual leaf area is summarized in Figure 2. In the following, the steps followed for building the models described in the flow chart are explained.

**Figure 3:**
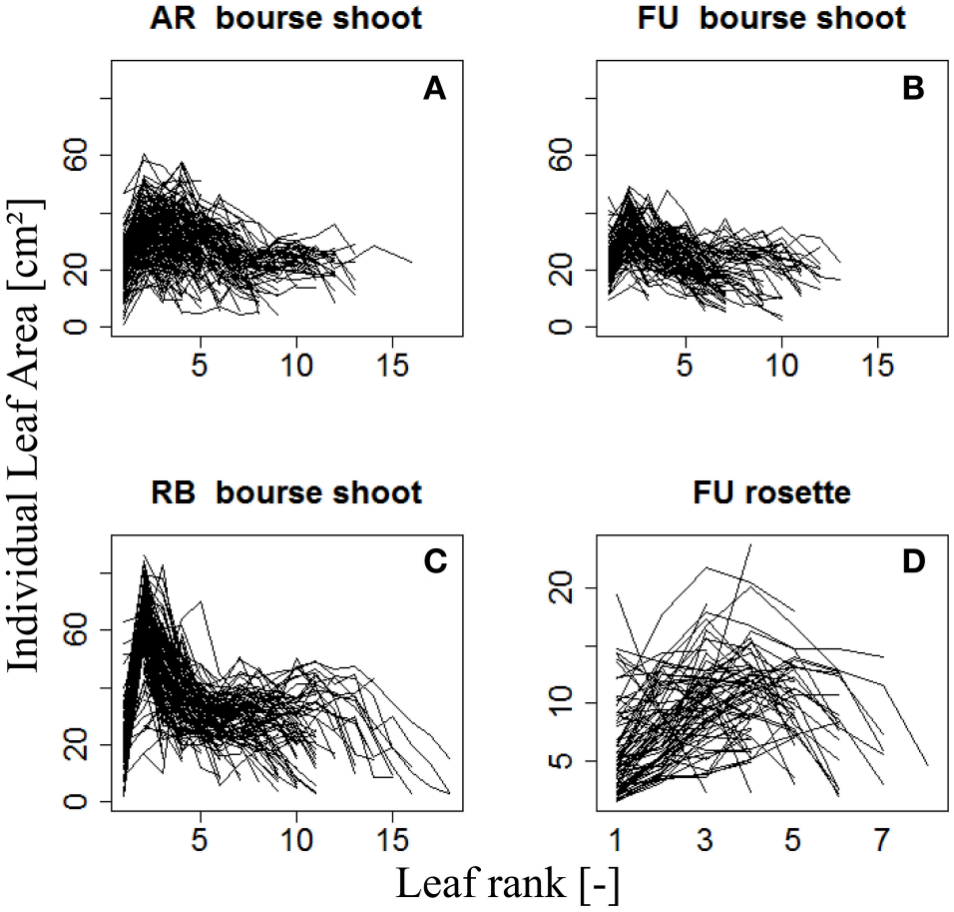
**Distribution of individual leaf area as a function of leaf rank of AR, FU, and RB bourse shoots (***N*** = 153, 97, and 112, respectively) (A–C)** and of FU rosettes (*N* = 80) **(D)**. Each line joins the individual leaf areas of a single shoot.

The variation of individual leaf areas of 153, 97, and 112 bourse shoots, respectively, of AR, FU, and RB, as a function of leaf rank is shown in (Figures 3A–C), and of 80 rosettes of FU considered to be fully grown shoots in (Figure 3D). Based on these observations, it was assumed that leaf size follows a rank-specific pattern typical for both bourse shoots and rosettes. In this study, we aimed to establish the pattern and parameterize it for each genotype and each type of shoot. However, the total leaf area of the shoot which can be calculated by integrating the sum of the areas of individual leaves of each rank seemed to be correlated with the number of leaves per shoot and the area of the biggest leaf (Figure 3). Furthermore, if this hypothesis would prove to be true, the result would confirm the existence of an allometric relation between the total leaf area on the one hand and the number of leaves and individual leaf area on the other. Moreover, if individual leaf areas follow a stable pattern along the shoot, this would mean that only one of these areas would be necessary as an input to the model, and that then the biggest leaf area would be an appropriate variable. Therefore, the assumption made and expressed in (Equation 3) implies that total leaf area (*TLA*) of the shoot is somehow related to the area of the biggest leaf (*A*_*max*_) and the number of leaves (*nl*).

(3) $$\begin{array}{l} TLA_{j,G}\;=\;\beta_{j,G}.\left(nl.A_{max}\right) \end{array}$$

ANOVA and Kruskal-Wallis tests were carried out on the selected data set in order to check if there is a significant difference of the parameter β_*j*,*G*_ among genotypes for a same shoot type. Groups of samples showing no significant difference were pooled and the training setestablished before were used in order to fix β_*j*,*G*_ using linear regressions between the variable *TLA* and the variable *nl.A*_*max*_.

Therefore, total leaf area of a shoot can be estimated by two variables: the area of the biggest leaf on the shoot (*A*_*max*_) and the number of leaves (*nl*) it bears. Thus, we made the assumption that the normalized individual leaf area [*N(A*_*R*_*)*, expressed as the ratio of the individual leaf area by the biggest leaf area on the shoot (Equation 4a)] is a function of the normalized leaf rank [*N(R)*], calculated as the ratio of the rank of the leaf divided by the number of leaves on the shoot (Equation 4b). However, we could observe that in bourse shoots, the leaf with the biggest area was by far the most often the second one (Table 1). Hence, in order to adjust a maximum of the curves describing *N(A*_*R*_*)* as a function of *N(R)*, the normalized rank for bourse shoots was calculated by dividing [the rank (*R*) minus 2] by the number of leaves (Equation 4c). Indeed, subtracting two from the rank of the biggest leaf (2) will result in a normalized rank which is always zero and will lead to a very good alignment of the curves. Besides, with this method, the entire curve is just shifted to the left without being stretched or compressed.

(4a) $$\begin{array}{r} N\left(A_{R}\right)=\frac{A_{R}}{A_{max}} \end{array}$$

(4b) $$\begin{array}{r} N_{RO}\left(R\right)=\frac{R}{nl} \end{array}$$

(4c) $$\begin{array}{r} N_{BS}\left(R\right)=\frac{R-2}{nl} \end{array}$$

**Table 1:** **Distribution of the leaves with the biggest area on bourse shoots with respect to their rank for AR, FU, and RB genotypes**.

**Genotype**	**Rank**					
	**1**	**2**	**3**	**4**	**5**	**6**
AR	0.65%	50.00%	26.62%	15.58%	5.84%	1.30%
FU	8.08%	68.69%	14.14%	3.57%	0.89%	_
RB	_	87.50%	8.04%	3.57%	0.89%	_

The models were established from predicting the normalized area of each leaf *N(A*_*R*_*)* as a function of the normalized leaf rank *N*_*j*_*(R)* by using the area of the biggest (in terms of leaf area) leaf (*A*_*max*_) on each shoot as a predictor of the other leaf areas. This hypothesis is expressed by Equation (5).

(5) $$\begin{array}{l} N\left(A_{R}\right)=f_{j,G}\left(N_{j}\left(R\right)\right) \end{array}$$

**Figure 4:**
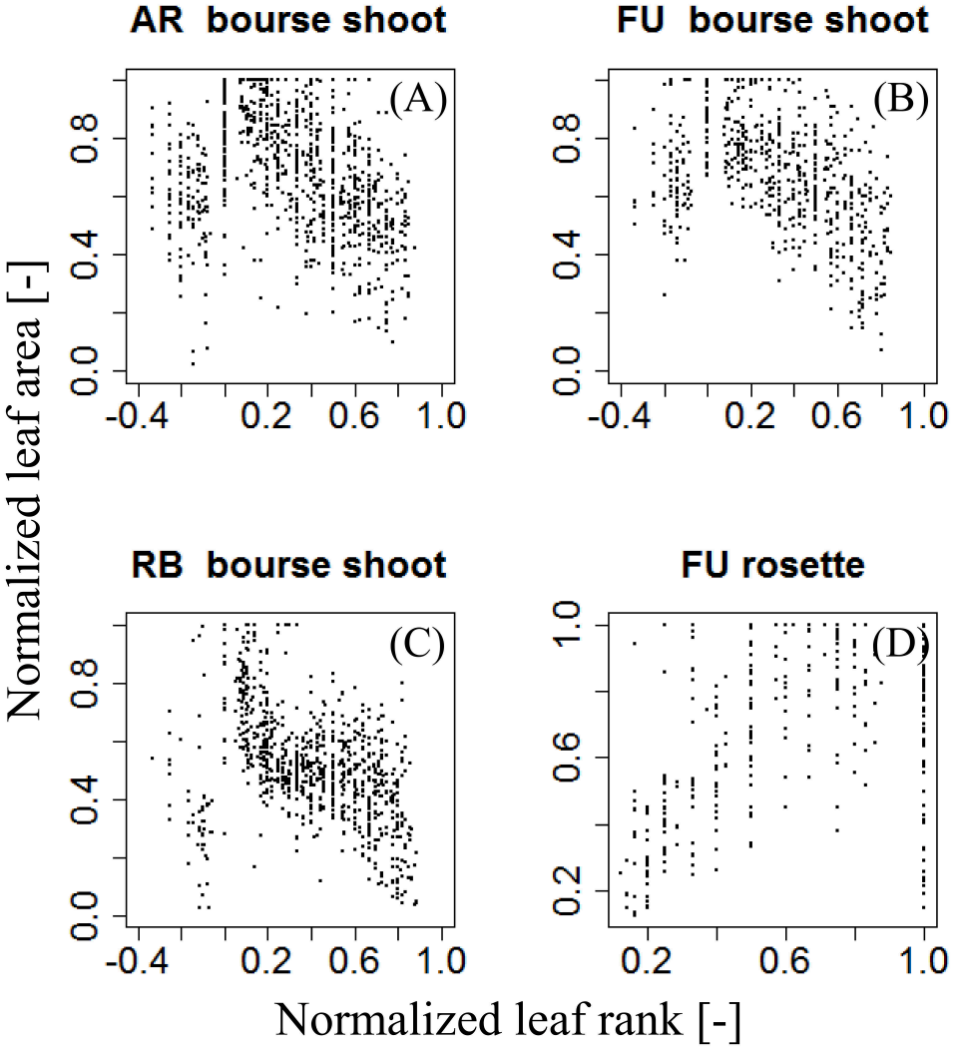
**Distribution of individual normalized leaf area plotted against normalized leaf rank of AR, FU, and RB bourse shoots (***N*** = 153, 97, and 112) (A–C)** and FU rosettes (*N* = 80) **(D)**.

For each type of shoot and each genotype, we looked for a function *f*_*j*,*G*_ that models the normalized area of each leaf on the shoot as a function of its normalized rank. *N(A*_*R*_*)* was plotted as a function of *N*_*j*_*(R)*, giving a certain pattern (Figure 4). We then tried to find a model that best described this relationship: a bilinear (broken stick) model and a Lorentz function. The first model requires five parameters while the second one only needs three. The Lorentz function has been successfully used by other workers (Buck-Sorlin, 2002; Evers et al., 2005, 2007; Gu et al., 2014) to predict leaf length in cereals and cotton. In this study, this function was chosen to model leaf areas of bourse shoots and rosettes.

(6) $$\begin{array}{l} f_{j,G}\left(x\right)=\frac{M}{1+\frac{{\left(x-x_{0}\right)}^{2}}{s^{2}}}\;;\;j\epsilon \left\{BS;RO\right\} \end{array}$$

The maximum is reached for *x* = *x*_0_, with *M* being the maximum value. *s* defines the slope of the curve of the function *f*_*j*,*G*_. For both bourse shoots and rosettes, *f*_*j*,*G*_ *(x)* corresponds to the normalized leaf area and *x* to the normalized leaf rank. *x*_0_ is the normalized rank of the leaf with highest area on the shoot [*N_j_(R_Amax_)*]. Consequently, the maximum of the function has the value of the normalized biggest area, i.e., *M* = *1* (Equation 7).

(7) $$\begin{array}{l} f_{j,G}\left(N_{j}\left(R\right)\right)=N\left(A_{R}\right)=\frac{1}{1+\frac{{\left(N_{j}\left(R\right)-N_{j,G}\left(R_{A_{max}}\right)\right)}^{2}}{s_{j,G}^{2}}};j\epsilon \left\{BS;RO\right\} \end{array}$$

After normalization for each type of shoot, Equations 7a and 7b are obtained:

(7a) $$\begin{array}{r} f_{BS,G}\left(N_{BS}\left(R\right)\right)=N\left(A_{R}\right)=\frac{1}{1+\frac{{\left(N_{BS}\left(R\right)\right)}^{2}}{s_{BS,G}^{2}}} \end{array}$$

(7b) $$\begin{array}{r} f_{RO,G}\left(N_{RO}\left(R\right)\right)=N\left(A_{R}\right)=\frac{1}{1+\frac{{\left(N_{RO}\left(R\right)-N_{RO,G}\left(R_{A_{max}}\right)\right)}^{2}}{s_{RO,G}^{2}}} \end{array}$$

Therefore, based on the fact that (i) the total leaf area (*TLA*_*j*,*G*_) of a shoot of type “*j*” is the sum of the individual areas *A*_*R*_ of leaves and (ii) its value depends on the pattern described by Equations 7a and 7b, the models (Equation 3) were derived as follows:

$$\begin{array}{l} \;TLA_{j,G}=\beta_{j,G}.(nl.A_{max})\\ \;\sum_{R\;=\;1}^{nl}A_{R}=\beta_{j,G}.(nl.A_{max})\\ \;\sum_{R\;=\;1}^{nl}\frac{A_{R}}{A_{max}}.\;\frac{1}{nl}=\beta_{j,G}\\ \;\sum_{R\;=\;1}^{nl}\frac{N(A_{R})}{nl}=\beta_{j,G}\\ \int \;f_{j,G}(N_{j}(R)).dN_{j}(R)=\beta_{j,G} \end{array}$$

$$\begin{array}{l} \{\begin{array}{l} \;\int_{\frac{-2}{nl}}^{\frac{nl\;-\;2}{nl}}\frac{1}{1+\frac{{\left(N_{BS}(R)\right)}^{2}}{s_{BS,G}^{2}}}.dN_{BS}(R)=\beta_{BS,G}\\ \int_{0}^{1}\frac{1}{1+\frac{{\left(N_{j}(R)-N_{RO,G}(R_{A_{max}})\right)}^{2}}{s_{RO,FU}^{2}}}.dN_{RO}(R)=\beta_{RO,G} \end{array} \end{array}$$

(8a,8b) $$\begin{array}{l} \{\begin{array}{l} {\left[s_{BS,G}.\text{atan}(\frac{N_{BS}(R)}{s_{BS,G}})\right]}_{\frac{-2}{nl}}^{\frac{nl\;-\;2}{nl}}=\beta_{BS,G}\\ {\left[s_{RO,G}.\text{atan}(\frac{N_{RO}(R)-N_{RO,G}(R_{A_{max}})}{s_{RO,G}})\right]}_{0}^{1}=\beta_{RO,G} \end{array} \end{array}$$

When solving Equation 8a used for the bourse shoot leaf area model, the only parameter to fix was *s*_*BS*,*G*_ and its different values were calculated as a function of the value of β_*BS*,*G*_ (which is unique for a same genotype and shoot type) and *nl* (from 1 to 18). Equation 8b used for parameterizing the rosette leaf area model does not take into account the number of leaves and it was solved using only β_*RO,G*_. However, in this second equation two parameters of the model [*s*_*RO,G*_ and *N*_*RO,G*_*(R*_*Amax*_*)*] had to be fixed. Both equations 8a and 8b were solved using the solver functionality of Microsoft Excel software (Microsoft, Redmond, WA, USA).

Starting from Equation 7, the model is expressed as:

(9) $$\begin{array}{r} N\left(A_{R}\right)\;=\;\frac{1}{1+\frac{{\left(N_{j}\left(R\right)-N_{j}\left(R_{A_{max}}\right)\right)}^{2}}{s_{j,G}^{2}}}\;;\;j\epsilon \left\{BS;RO\right\}\\ A_{R}=\frac{A_{max}}{1+\frac{{\left(N_{j}\left(R\right)-N_{j}\left(R_{A_{max}}\right)\right)}^{2}}{s_{j,G}^{2}}}\;;\;j\epsilon \left\{BS;RO\right\} \end{array}$$

(10) $$\begin{array}{r} A_{R}=\frac{A_{max}}{1+\frac{\frac{{\left(R-R_{A_{max}}\right)}^{2}}{nl^{2}}}{s_{j,G}^{2}}}\;;\;j\epsilon \left\{BS;RO\right\}\\ A_{R}=\frac{A_{max}}{1+\frac{{\left(R-R_{A_{max}}\right)}^{2}}{nl^{2}\cdot s_{j,G}^{2}}}\;;j\epsilon \left\{BS;RO\right\} \end{array}$$

By fixing the latter parameters, it was possible to calculate the normalized area of each leaf, using only the number of leaves on the shoot and the area of its biggest leaf. However, as the leaf area is not easily measurable non-destructively, further allometric relations are required for predicting leaf areas using variables that are more easily accessible. Leaf length and width seem to be the most obvious candidates for modeling the leaf area as we consider the leaf blade to be elliptical (Equation 11). Observations of leaves indicate that the length/width ratio is constant for a same genotype and shoot type (Equation 12). Assuming this is confirmed, only the variable “length of the leaf” (*L*) could be retained for calculating the individual leaf area. This assumption is stated as follows: (i) the ratio between the length (*L*) and the width (*W*) of a leaf is a constant parameter (*k*_*j*,*G*_*)* for the same shoot type (*j*) and the same genotype (*G*) and (ii) apple leaves have the shape of an ellipse (Freeman and Bolas, 1956). These two hypotheses are captured by Equations 11 and 12.

(11) $$\begin{array}{r} A=\pi \;\frac{L.W}{4} \end{array}$$

(12) $$\begin{array}{r} \frac{W}{L}=k_{j,G} \end{array}$$

The Shape of an ellipse is defined by its eccentricity *e*, i.e., the ratio between the distance from the center to a focus and the distance between that focus to a vortex (Equation 13a). Therefore, *k*_*j*,*G*_ can be expressed as a function of *e*_*j*,*G*_ (Equation 13b). If *e*_*j*,*G*_ is proven to be invariant for a same genotype and a same type of shoot, it could be used in the model as the constant for leaves on shoots of type *j* and of cultivar *G*; 0 < *k*_*G,j*_ ≤ 1

(13a) $$\begin{array}{r} e_{j,G}=\frac{\sqrt{L^{2}-W^{2}}}{L} \end{array}$$

(13b) $$\begin{array}{r} k_{j,G}=\sqrt{1-e_{j,G}^{2}} \end{array}$$

Therefore, *e*_*j*,*G*_ was calculated for each leaf used in this study using Equation 13a, as it is an indicator of the shape of the ellipse defined by the leaf. Then, a first test was done in order to check if there is a significant difference in *e*_*j*,*G*_ among the leaves of different ranks in a shoot within the same cultivar for each type of shoot. A second test was conducted to verify if there is a significant difference of *e*_*j*,*G*_ among the three genotypes for each type of shoot. Moreover, even if the leaf shape is genotype dependent, the environment can influence it during the last stages of leaf development (Tsukaya, 2004). Therefore, a test was done in order to check if there is a significant difference of *e*_*j*,*G*_ between 2014 (*N* = 323 and *N* = 159, for bourse shoots and rosettes, respectively) and 2015 (*N* = 385 and *N* = 363, for bourse shoots and rosettes, respectively) leaves of FU. Leaf data with no significant difference in *e* were pooled and the training set of the bearing spurs was used to fix *k*_*j*,*G*_ using a linear model.

Combining Equations (11) and (12), the following relation (14) is obtained:

(14) $$\begin{array}{l} A=\frac{\pi }{4}.k_{j,G}.L^{2} \end{array}$$

By combining Equation (10) and Equation (14), equation (15) is obtained.

(15) $$\begin{array}{l} A_{R}=\frac{\frac{\pi }{4}.k_{j,G}.L_{max}^{2}}{1+\frac{{\left(R-R_{A_{max}}\right)}^{2}}{nl^{2}\cdot s_{j,G}^{2}}};j\epsilon \left\{BS;RO\right\} \end{array}$$

In this way, *L*_*max*_ (the length of the biggest leaf on the shoot) and *nl* are the only two required input variables.

Using the models established we calculated individual leaf area and total leaf area on a shoot as the sum of the calculated individual leaf areas on the training set. Comparisons were made using the linear model between measured and calculated total leaf areas on each type of shoot and each genotype separately. Then, calibrations were made using a parameter α_*j*,*G*_ referring to the slope of the axis defined by measured total leaf area on a shoot (*TLA*_*j*,*G*_) as a function of calculated total leaf area on a shoot (C*TLA*_*j*,*G*_) of the training set. α_*j*,*G*_ was calculated for each type of shoot and each genotype separately.

The final model calculating individual leaf area is:

(16a) $$\begin{array}{r} A_{R}=\alpha_{BS,G}\frac{\frac{\pi }{4}.k_{BS,G}.L_{max}^{2}}{1+\frac{{\left(R-2\right)}^{2}}{nl^{2}.s_{BS,G}^{2}}} \end{array}$$

(16b) $$\begin{array}{r} A_{R}=\alpha_{RO,G}\frac{\frac{\pi }{4}.k_{RO,G}.L_{max}^{2}}{1+\frac{{\left(R-nl.N_{RO,G}\left(R_{A_{max}}\right)\right)}^{2}}{{nl}^{2}.s_{RO,G}^{2}}} \end{array}$$

The testing set was then used for calculating individual and total leaf areas. Using the testing data set, comparisons were made between measured and calculated data (i) for each established parameter, (ii) for individual leaf area and (iii) total leaf area on each shoot of each genotype using the linear model. *P*-values and coefficients of determination *R*^2^ were calculated in order to analyze and interpret the significance and the goodness of fit of the models. For rosettes, as the ranks of leaves on the bourse were not recorded in the 2014 experiment, comparisons between measured and calculated values of individual area were done only on data from 2015.

### Modeling the leaf area of vegetative shoots

**Figure 5:**
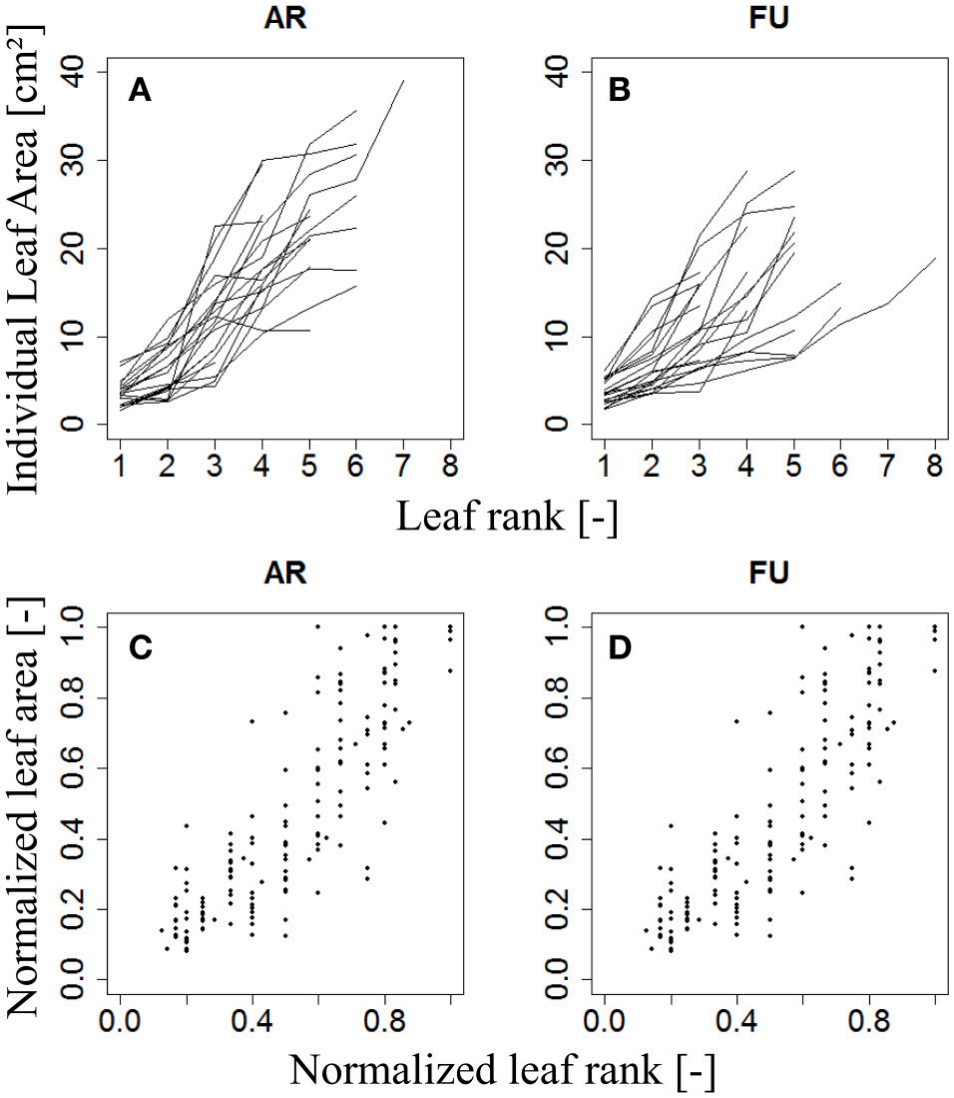
**Distribution of individual leaf area as a function of leaf rank (A,B)** and distribution of individual normalized leaf area as a function of normalized leaf rank **(C,D)** of AR and FU vegetative shoots (*N* = 20 vegetative shoots for each cultivar).

For modeling the leaf area of vegetative shoots, 20 vegetative shoots each of AR and FU were collected on July 3rd, 2015 when they were considered to be fully developed, then scanned and analyzed as described for bourse shoots and rosettes. Total leaf area on each vegetative shoot was calculated as the sum of areas of individual leaves. Leaf ranks on each shoot were recorded, and lengths and dry weights of the shoots and the leaves measured. The variation of individual leaf areas of the 20 vegetative shoots of AR and FU as a function of leaf rank is shown in (Figures 5A,B): leaf area increases with rank for both genotypes. However, the pattern is heterogeneous when based on absolute leaf length measurements and ranks. We therefore normalized both leaf areas (Equation 4a) and ranks (Equation 4b). On visual inspection, the distribution of *N(A*_*R*_*)* as a function of *N(R)* on vegetative shoots of both genotypes (Figures 5C,D) seems to describe a linear pattern (Equation 17). We made the assumption that *A*_*R*_ is proportional to the square of leaf length *L_R_*^2^ as for bourse shoots and rosettes (Equation 14). Eccentricity (*e*_*VS*,*G*_) of the ellipse was calculated for each leaf and comparison tests were made between the two genotypes, then *k*_*VS*,*G*_ (ratio between width and length of the leaf blade) was fixed for the model. Afterwards and using the previously fixed *k*_*VS*,*G*_, a parameter *p* which describes the slope of the curve was calculated for each leaf using Equation 17 and a statistical comparison was carried out between the two cultivars in order to check if they were different with respect to *p*. Based on this comparison, *p* was fixed using a linear regression model. The vegetative shoot model (Equation 18) is a descriptive model allowing calculating the individual leaf areas of AR and FU vegetative shoots using only two variables, *L*_*max*_ and *nl*. A regression test was carried out using the linear model between the calculated individual areas and the measured ones.

(17) $$\begin{array}{r} N\left(A_{R}\right)=p.N\left(R\right) \end{array}$$

(18) $$\begin{array}{r} \frac{A_{R}}{A_{max}}=p.\frac{R}{nl}\\ A_{R}=p.\frac{R}{nl}.A_{max}\\ A_{R}=p.\frac{R}{nl}.\frac{\pi }{4}.k_{j,G}.L_{max}^{2} \end{array}$$

### Modeling length of spur internodes

**Figure 6:**
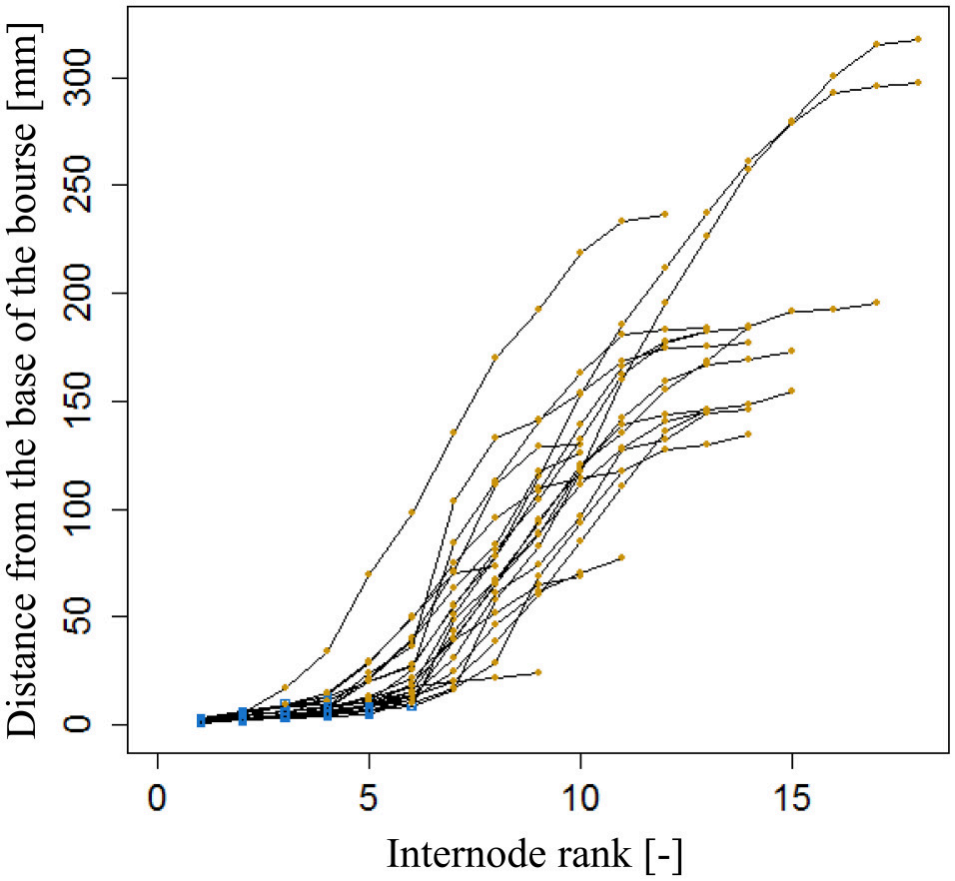
**Cumulated length of bourse and bourse shoots, expressed as the distance from the base of the bourse to the point of insertion of a bourse or a bourse shoot leaf of internode rank 1–18 in FU [***N*** = 24, continuum: bourse (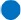) - bourse shoot (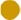)]**.

The internode lengths of 20 spurs of “FU” were measured in the orchard in July 2016. As the bourse internodes are very short, we assigned to each one the mean value of the bearing spur calculated as the ratio of “Length of the bourse”/“Number of internodes on the bourse.” For each spur, a “cumulative shoot length” *D*_*I*_ between the base of the bourse (rosette) and the node (leaf insertion point) for each bourse and bourse shoot leaf was measured. For bourse shoot nodes, this cumulative shoot length is equal to the sum of the lengths of the rosette internodes from the base of the bourse to the node on which is inserted the bourse shoot (*BL*) and the sum of the internode lengths from the base of the bourse shoot to the considered node. The rank of each internode, on each spur, was also recorded from the first rosette leaf to the last bourse shoot leaf, assuming that the rank of the first internode of the bourse shoot has the value of the rank of the bourse internode on which it is inserted, plus one. Among the 20 spurs, 16 were bearing only one bourse shoot and the remaining ones were bearing two bourse shoots. Plotting this cumulative shoot length *D*_*I*_ as a function of cumulative (bourse and bourse shoot) rank *R*_*I*_ corresponded to a logistic pattern for each spur (Figure 6). However, the pattern seemed to be dependent upon the length of the shoot (bourse plus bourse shoot). Therefore, normalized cumulative internode lengths [*N(D*_*I*_*)*] between each leaf base and the base of the bourse (Equation 19a) and normalized rank [*N(R*_*I*_*)*] (Equation 19b) were calculated assuming that the bourse and the bourse shoot are the same shoot unit (and that, therefore, the bourse shoot is a sylleptic extension of the bourse). The normalized cumulative shoot length was calculated as a function of the sum of *BL* and the bourse shoot length (*BSL*).

(19a) $$\begin{array}{r} N\left(D_{I}\right)=\frac{D_{I}}{BL+BSL} \end{array}$$

(19b) $$\begin{array}{r} N\left(R_{I}\right)=\frac{R_{I}}{nl} \end{array}$$

Afterwards, the parameters *qi* and *si* of the internode model were fixed by fitting a logistic model (Equation 20) to the distribution of the *N(D*_*I*_*)* as a function of *N(R*_*I*_*)*.

(20) $$\begin{array}{l} N(D_{I})=\frac{1}{1\;+\text{ exp }\left(\frac{qi-N\left(R_{I}\right)}{si}\right)} \end{array}$$

Once established, this descriptive model would estimate the cumulative shoot length of each leaf insertion point of rank *R*_*I*_ on the bourse or the bourse shoot from the bottom of the bourse providing 3 input variables: *nl*, (*BL* + *BSL*) and *R*_*I*_ (Equation 21).

(21) $$\begin{array}{l} D_{I}=\frac{1}{1\;+\text{ exp }\left(\frac{qi-\frac{R_{I}}{nl}}{si}\right)}.(BL+BSL) \end{array}$$

### Statistical analyses

Statistical analyses were done using R Studio software version 0.98.1062.0 running R version 3.2.2. and the statistical computing environment of the R-package “agricolae,” version 1.2-2. Normality of data used for each analysis was tested on residuals using the Shapiro-Wilk test for data sets with more than 50 samples per group or sample distributions considered to be normal (*P* > 0.05); ANOVA tests were used to check if there was a significant difference among groups (*P* < 0.05). Otherwise differences between groups were tested with the Kruskal-Wallis test (*P* < 0.05), or both ANOVA and Kruskal-Wallis. Fit of data to selected models was checked using the *lm* function in *R* software for linear distribution models. The logistic model was parameterized using the *SSlogis* function in *R* software and coefficients of determination (*R*^2^) and root mean squared errors (RMSE) were calculated to test the fit of the model.

## Results

### Modeling the leaf area of bourse shoots (BS)

**Figure 7:**
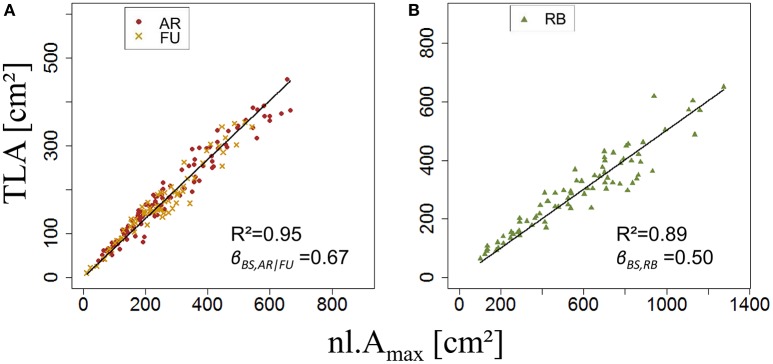
**Parameterization of β_***BS,G***_ (***N*** = 180 and 80 bourse shoots, respectively; A,B)**.

ANOVA made on β_*BS*,*G*_ of bourse shoots showed there was no significant difference between AR (mean β_*BS,AR*_ = 0.71) and FU (mean β_*BS,FU*_ = 0.70). However, bourse shoots of both AR and FU were significantly different from RB with respect to β_*BS*,*G*_ (mean β_*BS,RB*_ = 0.53). Therefore, the same value of β_*BS*,*G*_ was used for the training data set of AR and FU bourse shoots, while for RB bourse shoots a separate training data set was used, thus necessitating the parameterization of two linear regression models (Figures 7A,B). For both groups, the linear model was significant (*P* < 2.10^−16^). The coefficients of determination for both the AR|FU model and the RB model were sufficiently high (*R*^2^ = 0.95 and *R*^2^ = 0.89) to support the assumption that the values of β_*BS*,*G*_ (β_*BS,AR*/*FU*_ = 0.67 and β_*BS,RB*_ = 0.50) were robust.

Using the values of β_*BS*,*G*_ Equation 8a was solved and *s*_*BS,AR*|*FU*_ and *s*_*BS,RB*_ were established as a function of the number of leaves on the bourse shoot. The number of leaves recorded on bourse shoots sampled varied from 1 to 16, 13 and 18, respectively, for AR, FU and RB. Thus 18 values for *s*_*BS*,*G*_ were established for each one of the two groups of genotypes (Table 2).

**Table 2:** **Parameterization of ***s***_***BS,G***_**.

**NL**	**AR & FU**	**RB**
1	2.13	1.48
2	0.70	0.43
3	0.38	0.23
4	0.35	0.22
5	0.36	0.22
6	0.38	0.23
7	0.40	0.24
8	0.42	0.25
9	0.44	0.25
10	0.46	0.26
11	0.48	0.27
12	0.49	0.28
13	0.51	0.29
14	0.52	0.30
15	0.53	0.31
16	0.54	0.31
17	0.55	0.32
18	0.55	0.32

*e*_*BS*,*G*_ was significantly different among genotypes (*p* < 2.2.10^−16^), with means of *e*_*BS*,*G*_ equal to 0.79, 0.76, and 0.74 for AR, FU and RB, respectively. ANOVA tests showed no significant differences among the shapes of leaves of different ranks on a bourse shoot (*e*_*BS*,*G*_). However, leaves above rank 14 in AR and leaves above rank 15 in RB seemed to indicate a significant difference, but based on the weak frequencies of these leaf ranks (only one bourse shoot of AR and 15 bourse shoots of RB having more than 13 and 15 leaves, respectively), it was decided not to take into account this difference. Furthermore, *e*_*BS,FU*_ was not significantly different between 2014 and 2015 (*p* = 0.59). Therefore, it was considered that the impact of the factor year was not significant.

**Figure 8:**
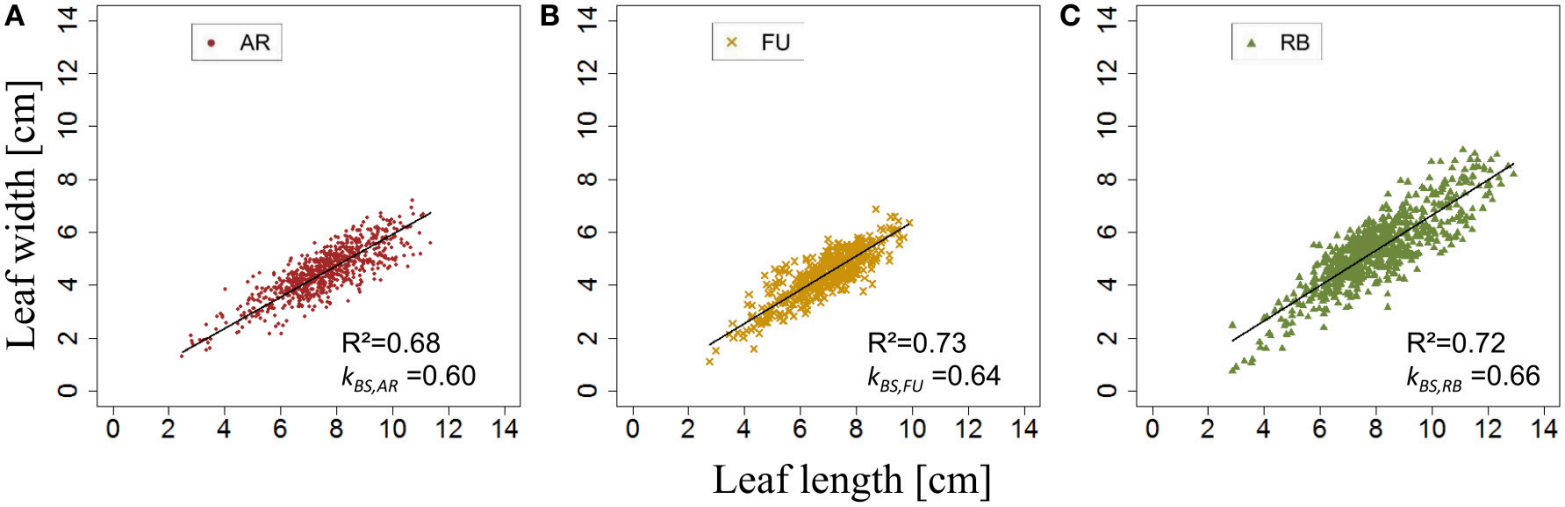
**Parameterization of ***k***_***BS,G***_ (***N*** = 763, 465, and 655 bourse shoot leaves, respectively; A–C)**.

*k*_*BS*,*G*_ was fixed for AR, FU and RB using linear regression models on the training set of each genotype (Figures 8A–C) and the leaf length of each genotype was significantly correlated with its width (*P* < 2.10^−16^ for all the genotypes' models), with coefficients of determination of 0.68, 0.73, and 0.72 for AR, FU and RB, respectively. *k*_*BS*,*G*_ values were fixed to 0.60, 0.64, 0.66 for AR, FU and RB, respectively.

**Figure 9:**
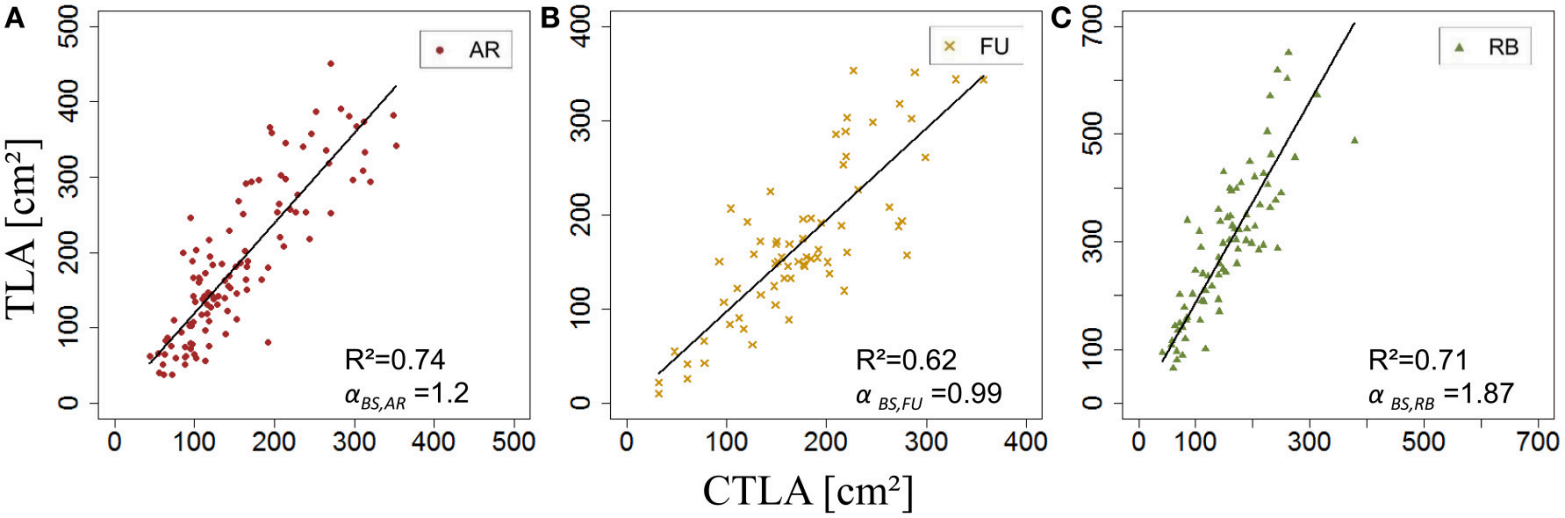
**Calibration of the bourse shoot leaf area model: parameterization of α_***BS,G***_ (***N*** = 114, 66, and 80 bourse shoots, respectively; A–C)**.

**Figure 10:**
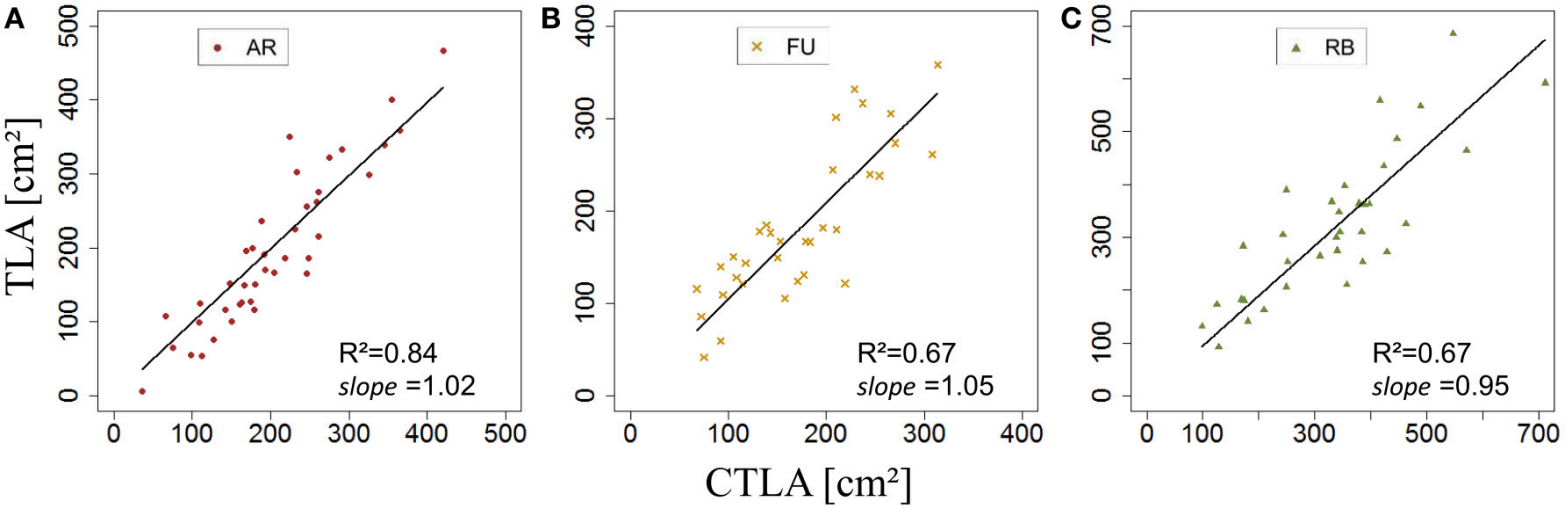
**Testing of the bourse shoot leaf area model (***N*** = 40, 33, and 34, bourse shoots respectively; A–C)**.

Once *s*_*BS*,*G*_ and *k*_*BS*,*G*_ were fixed, all leaf areas of the bourse shoot training set were calculated using Equation 15, with the rank of the leaf and the variables *L*_*max*_ and *nl* of each bourse shoot as input. Total leaf area on each shoot was calculated as the sum of individual leaves. Calculated and measured total leaf areas (*CTLA*_*BS*,*G*_ and *TLA*_*BS*,*G*_) of the bourse shoots were compared for each genotype using a linear regression where the slope α_*BS*,*G*_ of each regression model of *TLA*_*BS*,*G*_ as a function of C*TLA*_*BS*,*G*_ was expected to be equal to 1 if the calculated and the measured area were identical. The slope parameter was equal to 1.2, 0.99 and 1.87 for AR, FU and RB, respectively (Figures 9A–C), which led us to assume that the parameter α_*BS*,*G*_ was genotype dependent. Therefore, values of α_*BS*,*G*_ fixed using the training set were retained for calibrating the model. The model defined in Equation 15 and built for bourse shoots including the three parameters (*s*_*BS*,*G*_, *k*_*BS*,*G*_ and α_*BS*,*G*_) and using the variables *L*_*max*_ and *nl* for the prediction of individual leaf areas was used to calculate individual areas for each leaf of the testing set and the comparison with measured individual data was conducted using a linear model. In the testing set, though linear models of calculated individual leaf area as a function of measured individual leaf area were significant for all genotypes (*P* < 2.10^−12^), the coefficients of determination were very small (*R*^2^ = 0.17, 0.20, 0.30 for AR, FU and RB, respectively). In contrast to this, linear regression modeling of *TLA*_*BS*,*G*_ as a function of C*TLA*_*BS*,*G*_ yielded significant results for all genotypes (*R*^2^ = 0.84, 0.67 and 0.64; slope = 1.02, 1.05 and 0.95 for AR, FU and RB, respectively; Figures 10A–C).

### Modeling the leaf area of rosettes (RO)

**Figure 11:**
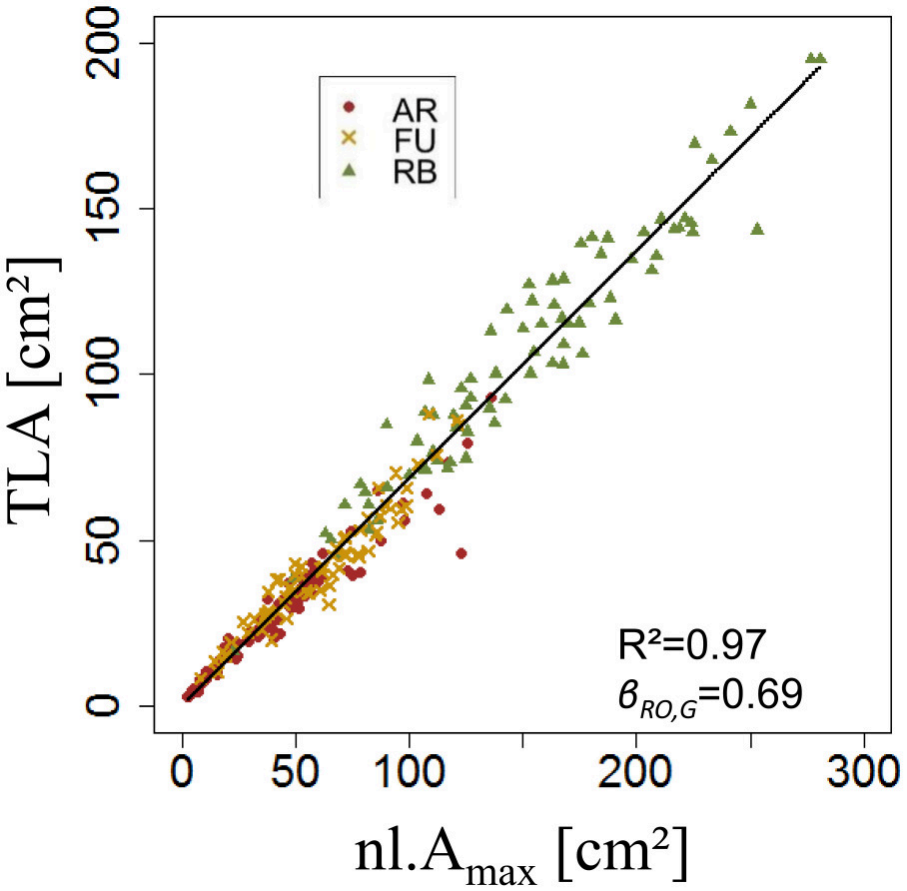
**Parameterization of β_***RO,G***_ (***N*** = 232 rosettes)**.

Both ANOVA and Kruskal-Wallis test showed there was no significant difference between the three genotypes with respect to β_*RO,G*_. Thus β_*RO,G*_ was fixed to 0.69 using a linear regression model on the training set of rosette data of bourses sampled before harvest of the three genotypes (Figure 11A). The model was significant (*P* < 2.10^−16^) and the coefficient of determination was high (*R*^2^ = 0.97). Afterwards, the values of parameters *s*_*RO,G*_ and *N*_*RO,G*_*(R*_*Amax*_*)* were fixed to 0.38 and 0.63, respectively.

*e*_*RO,G*_ was significantly different among genotypes (*P* = 3.46.10^−8^ < 0.05), with means of *e*_*RO,G*_ equal to 0.65, 0.66 and 0.68 for AR, FU and RB, respectively. When comparing *e*_*BS*_ of different ranks on rosettes of FU sampled in 2015, both ANOVA and Kruskal –Wallis test showed there were no significant differences among the shapes of leaves of different ranks on the bourse. *e*_*RO,FU*_ did in fact significantly vary between the 2 years (*P* = 2.162.10^−7^).

**Figure 12:**
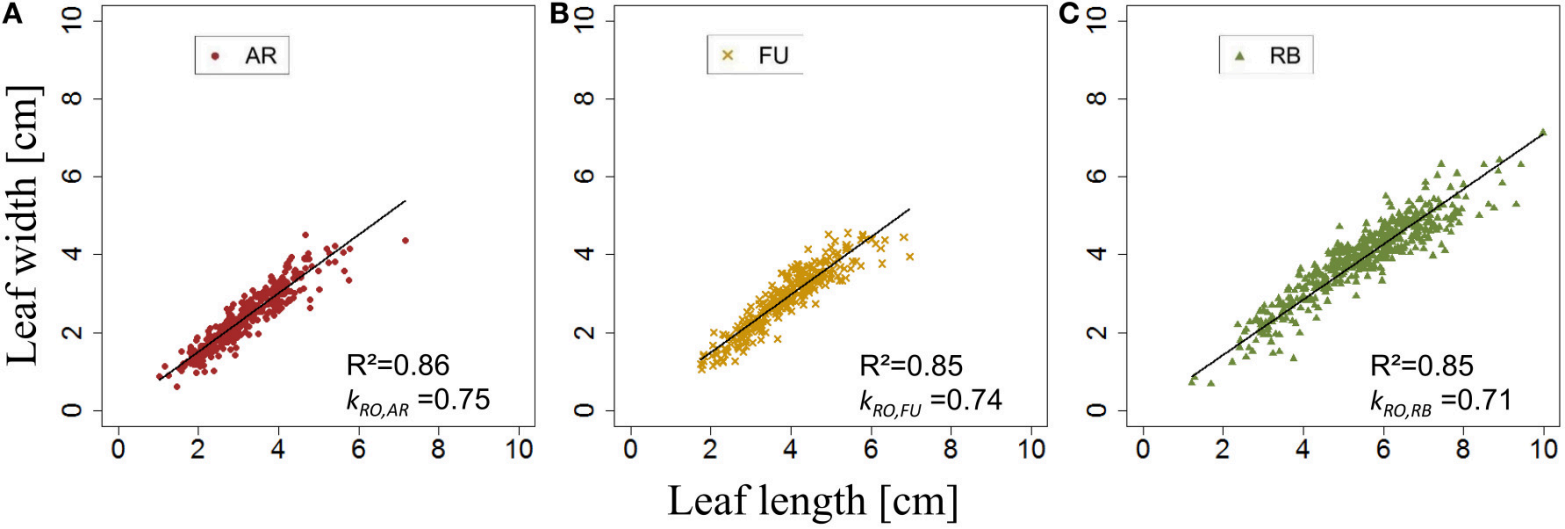
**Parameterization of ***k***_***RO,G***_ (***N*** = 375, 303, and 475 rosette leaves, respectively; A–C)**.

**Figure 13:**
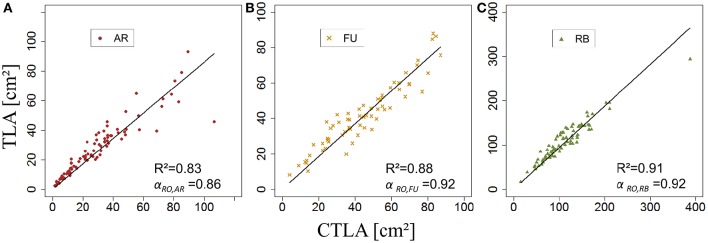
**Calibration of the rosette leaf area model: parameterization of α_***RO,G***_ (***N*** = 87, 67, and 78 rosettes, respectively; A–C)**.

**Figure 14:**
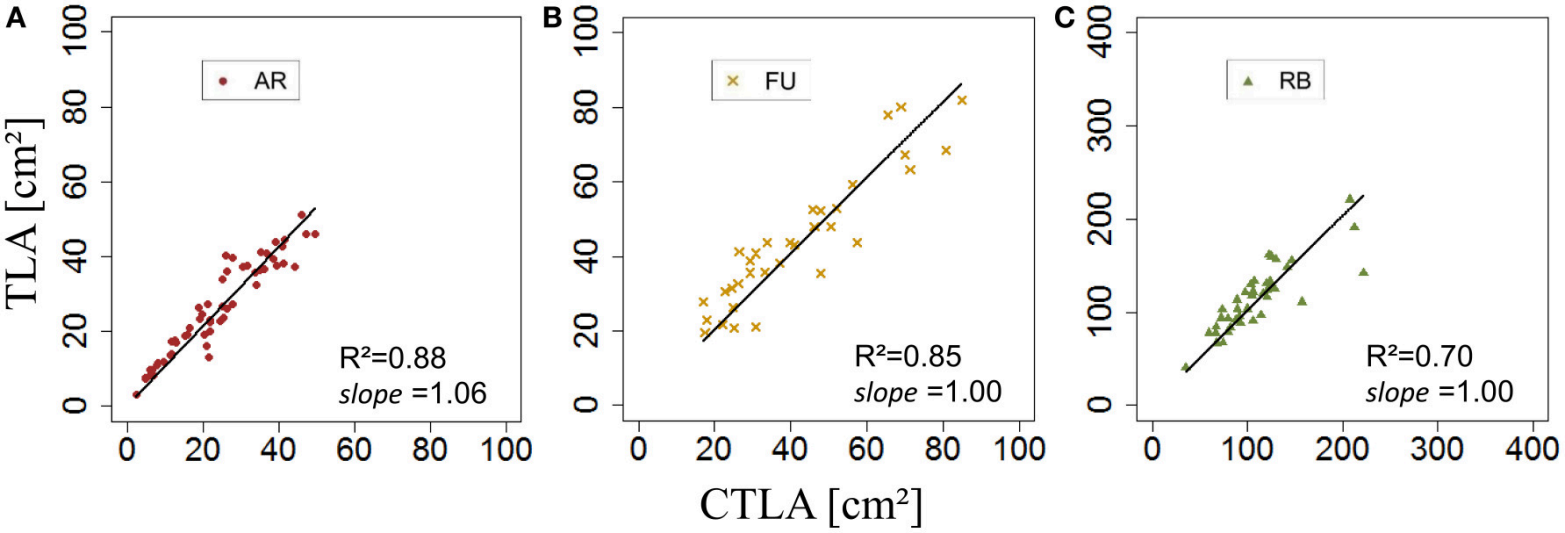
**Testing of the rosette leaf area model (***N*** = 57, 33, and 40 rosettes, respectively; A–C)**.

*k*_*RO,G*_ was established for AR, FU, and RB using linear regression models on the training set of each genotype (Figures 12A–C) and the leaf lengths of each genotype were significantly correlated with leaf width (*P* < 2.10^−16^ for all the genotypes' models) with *R*^2^ = 0.86, 0.85 and 0.85 for AR, FU and RB, respectively. *k*_*RO,G*_ values were fixed to 0.75, 0.74 and 0.71 for AR, FU and RB, respectively. Regression models of *TLA*_*RO,G*_ as a function of C*TLA*_*RO,G*_ conducted on the training data set showed a significant relationship (*P* < 2.10^−16^) for the three genotypes with *R*^2^ of 0.83, 0.88 and 0.91 for AR, FU and RB, respectively. However, the C*TLA*_*RO,G*_ were bigger than the *TLA*_*RO,G*_ as can be seen from the slopes. Therefore, their values were assigned to α_*RO,G*_ (0.86, 0.92, 0.92, respectively) to calibrate the rosette model (Figure 13). After that, individual and total leaf areas for each rosette were established for the testing set. The linear regression model of measured individual leaf area as a function of calculated individual leaf area showed a significant relationship (*P* < 2.10^−16^) for FU samples of 2015 (*R*^2^ = 0.52). The slope of the regression model (ratio between measured and calculated leaf area) was equal to 0.92. Linear models simulating *TLA*_*RO,G*_ as a function of C*TLA*_*RO,G*_ were all significant (*P* < 2.10^−16^). The predictive models (Figure 14) had high coefficients of determination (0.88, 0.85 and 0.70 for AR, FU and RB, respectively) and the slopes were equal to 1 for the three genotypes (1.06, 1 and 1, respectively).

### Modeling the leaf area of vegetative shoots (VS)

Neither ANOVA nor Kruskall-Wallis tests showed any significant difference in *e*_*VS*,*G*_ between AR and FU (*P* = 0.54 and 0.49, for each test, respectively). Thus, *k*_*VS*,*G*_ was fixed (*k*_*VS*,*G*_ = 0.56) using a linear regression model using data of both genotypes. The regression model showed a significant relationship between leaf length and width (*P* < 2.10-16) and *R*^2^ was equal to 0.79. Besides, neither ANOVA nor Kruskal-Wallis test showed any significant difference in the variable *p* between genotypes AR and FU. The linear model used to fix *p*_*VS*,*G*_ (*p*_*VS*,*G*_ = 0.95) was significant (*P* < 2.10^−16^) with *R*^2^ = 0.83.

### Modeling internode lengths of spurs

**Figure 15:**
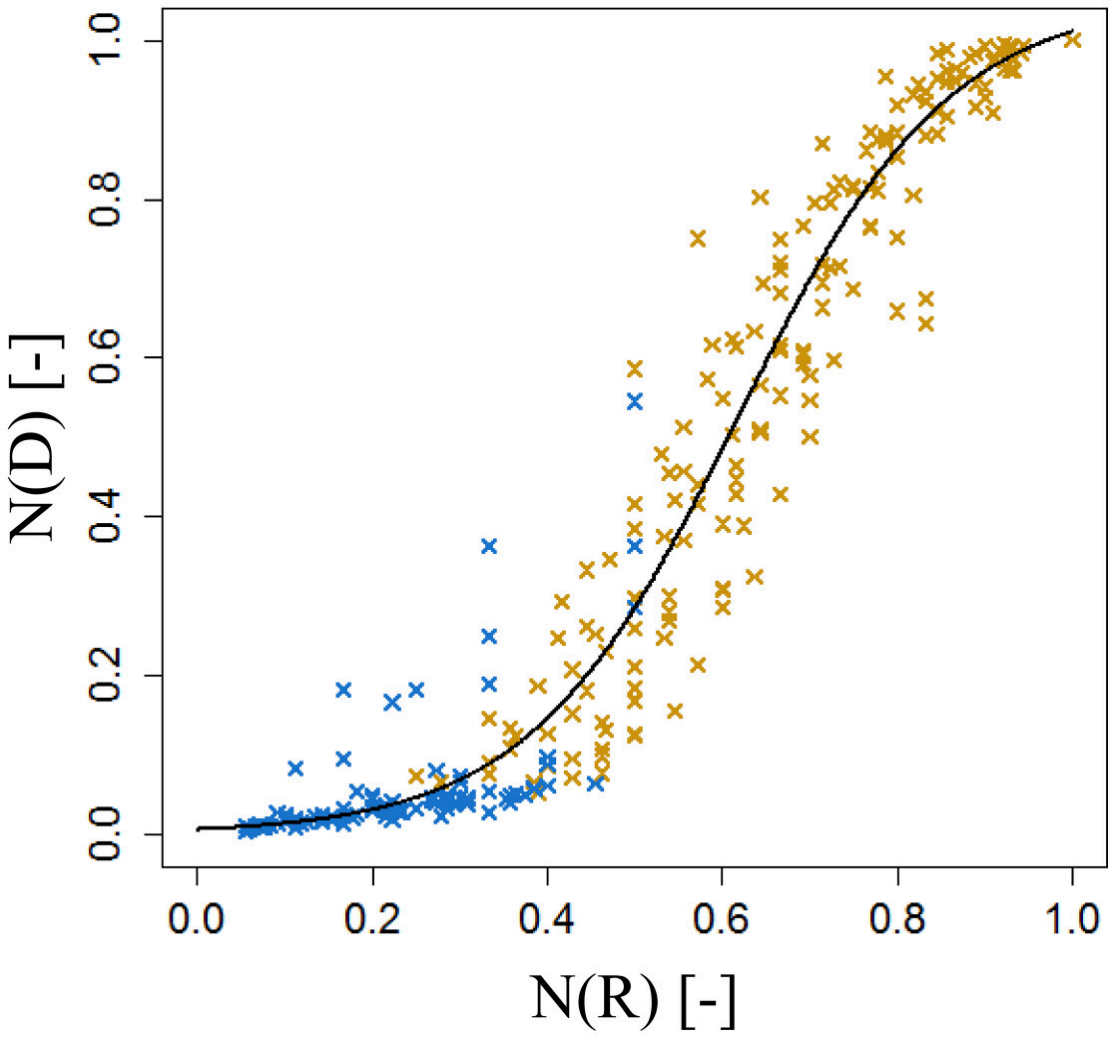
**Parameterization of the cumulative shoot length model on FU spurs**. *N(R*_*I*_*)*, normalized rank; *N(D*_*I*_*)*, normalized cumulative shoot length from the base of the bourse [*N* = 24, continuum: bourse (x) – bourse shoot (x)].

Parameterization of the internode model fixed the value of *qi* to 0.62 and that of *si* to 0.12 (Figure 15). Both the coefficient of determination and the RMSE indicated a very good fit of the model to the distribution of measured data (*R*^2^ = 0.96; RMSE = 0.08). Indeed, the model exhibited a common pattern involving bourse and bourse shoot internodes. Moreover, the model showed that rosette internodes correspond to the first part of the logistic equation (exponential phase), while bourse shoot internodes belong to the second part (linear phase).

## Discussion

Several studies show the determinism of plant architecture traits (Lauri and Trottier, 2004; Kahlen and Stützel, 2007; Massonnet et al., 2008). In this study we assumed that the distribution of normalized individual leaf area as a function of normalized rank is described by a similar pattern regardless of shoot size and, therefore, that the total leaf area of a shoot is proportional to the product of the leaf number times the area of the biggest leaf. Spann and Heerema (2010) also showed relationships between shoot leaf area and the product of the number of leaves and length of longest leaf, for different species. However, the models presented here were conceived with the aim to link parameters that have the same unit (i.e., cm^2^), both being expressions of surface. This is also the reason why in our final model, which only takes into account leaf lengths and numbers, leaf length is expressed as the square of itself. What is more, the linear model linking measured shoot total leaf area (*TLA*) with the product of leaf number (*nl*) and area of the biggest leaf (*A*_*max*_), exhibits a larger coefficient of determination than the regression between *TLA* and the product of *nl* and the length of the longest leaf (*L*_*max*_) (data not shown). Although the predictive capacity of our models with respect to individual leaf area was not optimal, the model was nevertheless based on a very good prediction of the total shoot leaf area. Indeed, in the case of the rosette and the bourse shoot, we have chosen to construct our model as a function of the parameter β which is described by the slope of *TLA* versus *nl.A*_*max*_, and not by parameterizing a Lorentz function, on the distribution of the point cloud which describes the normalized individual leaf surface as a function of the normalized rank. This choice was made on the one hand because of the absence of a particular pattern and therefore, the scatter of data points was considered to be too big; on the other hand, because of the proven robustness of β which we wanted to conserve for the development of the model. Besides, it is not unlikely that the fact that we had to use a calibration parameter (α) for each genotype and each shoot type (rosette and bourse shoot) is due to the way the point cloud deviates from the chosen Lorentz equation according to the genotype. Furthermore, even if the prediction of individual leaf area is not ideal, it is still faithful to an existing pattern of distribution of individual leaf area as a function of rank. Similar patterns were described for wheat (Pararajasingham and Hunt, 1996; Bos and Neuteboom, 1998; Hotsonyame and Hunt, 1998) and for rice (Tivet et al., 2001; Zhu et al., 2009). As an alternative to the Lorentz equation, we tested a bilinear equation with five parameters. However, apart from the higher number of parameters compared to Lorentz, this equation also yielded a higher RMSE.

Furthermore, the models developed were thought to go beyond predicting only shoot total leaf area. Indeed, the prediction of individual leaf area is more relevant if we aim to use this in an FSPM because in this approach parameterization takes place at the organ scale. Moreover, unlike the rosette which can be considered a compact functional unit in terms of leaf area, the bourse shoot usually exhibits a more open structure conveying more importance to the individual leaf with respect to light interception or exposure to liquids or dusts (in the case of chemical treatments: Dekeyser et al., 2013; Duga et al., 2015). However, even in a rosette, individual leaf area and its distribution along the shoot will determine the mutual shading of leaves and, therefore, light interception. Furthermore, in an FSPM, the position, shape and orientation of each organ are required as an input. The model proposed in this study is an attempt to optimize and simplify the reconstruction of branch architecture which is needed as an input for many FSPMs. As pointed out by Fisher (1984), the leaf surface of the crown is determined by phyllotaxis of the shoot, leaf orientation, clustering of new leaves on short shoots, internode lengths, and distribution of leaves along a branch (Fisher, 1984). Therefore, besides predicting leaf area (and its distribution along the shoot) our model also integrated internode lengths distribution and phyllotactic leaf angles (data not shown).

The data set used for the establishment of the leaf length model for bourse shoots was based on leaves sampled from shoots from a wide range of dates. This was justified as we determined the time (in GDD) from which onwards shoot leaf areas did no longer differ significantly from final leaf areas in the three different genotypes. This time roughly coincides with the date found by Da Silva et al. (2014), who indicated the 30th of June of each year as the end of annual primary shoot extension for most shoot types.

We found that the shape of rosette leaves, independent of genotype, was always more circular than that of bourse shoots which is not surprising as rosette leaves are preformed (Lauri, 2007). In this study, AR exhibited extreme values for the parameter that describes leaf shape (*k*), with bourse shoot leaves being much more elongated (*k*_*BS,AR*_ = 0.60) than rosette leaves (*k*_*RO,AR*_ = 0.75). This was in sharp contrast to RB where bourse shoot leaves (*k*_*BS,RB*_ = 0.66) and rosette leaves (*k*_*RO,RB*_ = 0.71) were much more similar in shape. FU was intermediary between AR and RB. An interesting observation made in this study was that the descriptive model of internode lengths could be optimized if we considered the bourse rosette and the bourse shoot as one single continuous morphogenetic unit. The bourse shoot being a sylleptic extension of the bourse, it in fact represents the continuation of the rosette. We can thus state that the base of the rosette up to the insertion point of the bourse shoot, plus the bourse shoot itself, is forming a unit that is characterized by a basal zone with short internodes, followed by a median zone with longer internodes (base of the bourse shoot), and a subapical zone with short internodes (Buck-Sorlin and Bell, 2000). This tentative conclusion gives rise to the idea that leaf length prediction could be further improved by considering leaf length distribution of the continuum of rosette plus bourse shoot instead of treating the two shoots separately. It would furthermore be interesting to see if there are allometric relations between the length of an internode and the area of the leaf that is inserted on it, given the fact that both have been produced by the vegetative meristem during the same developmental event. According to Da Silva et al. (2014) it is unlikely that leaf area changes independently from internode length, as metamers exhibit a strong allometry (Fisher, 1986).

In the present study we chose to split our data into training and testing sets: the first one was used to calibrate our model while the latter was employed to test it. Admittedly, some aspects of this procedure are arguable: first of all, it could be claimed that the splitting (two thirds training to one third testing) was arbitrary. However, enough data was available to have sufficiently large training and testing sets and more data was needed for calibration than for testing. Secondly, we confounded the FU data sets of the two experimental years (2014 and 2015)—whereas for AR and RB only data of 2014 was available—and randomly distributed the data into the two sets, instead of using 1 year's data set for calibration and the other one for testing. However, the data sets revealed no significant inter-annual variation, except for the shape of rosettes in FU. As the 2015 data set was smaller than the one from 2014 and contained data only for FU, future testing with independent data sets has to be conducted to further prove the robustness of our models. Thirdly, as an alternative we could have neglected model testing and just have included a description of the variance (as was in fact done for the leaf area of vegetative shoots in this study). However, this would have meant neglecting the clearly robust and stable patterns that emerged among some of the meristic (leaf number) and continuous (length of the biggest leaf, total and individual leaf area) traits. In this study, the use of a test data set was necessary to check the fit of the final model. Indeed, the final model cumulates all the errors of the intermediate steps of parameterization and calibration, and the testing allowed us to quantify this cumulated error.

An accurate prediction of total shoot leaf area for each shoot type, and of the distribution of single leaf area as a function of leaf rank, in connection with individual internode length, are the first steps toward the faithful reconstruction of architecture, to be used in an FSPM to compute light interception or even to estimate the percentage of hidden surfaces in simulations of the efficiency of pesticide spray applications (Dekeyser et al., 2013; Duga et al., 2015). With respect to light interception, leaf area and internode length are in fact the two most important traits contributing to plant architecture whereas branching angle seems to have only little impact on this functional trait (Han et al., 2012; Da Silva et al., 2014). It also appears that in order to explain leaf distribution within the tree the shoot scale is the most appropriate one (Da Silva et al., 2014). This justifies the choice we have made in this study, namely to concentrate on the modeling of leaf area distribution along different shoot types and to neglect divergence angles. As the spatial distribution of leaves in apple trees is not random (Da Silva et al., 2014) but rather characterized by a certain clumpiness (also reported for other tree species by Cohen et al., 1995; Chen et al., 1997; Da Silva et al., 2008), the next step in the reconstruction of branch architecture has to be to position and orient the different shoot types. In doing so, it has to be considered that the proportion of long and short shoots differs among genotypes (Lauri et al., 1995). The genotypes used in this study exhibited such a numerical variability at the scale of the first-order branch, with bourse shoots in AR being much more important than in RB (data not shown). The analysis of the distribution of shoots within the branch will be the subject of a follow-up study (Bairam et al., unpubl.). In any case, it is thus necessary to count the number of shoots of the different types or better still, create a topological map of the branch and its different organs (see Buck-Sorlin and Bell, 2000, for an example) in order to finalize the reconstruction of branch architecture.

## Conclusions and perspectives

In the present study, we provided a new model allowing the reconstruction of the initial branch architecture as an input for a Functional Structural Plant Model of apple, with an emphasis on the prediction of leaf area at the shoot and leaf scale, using allometric relations among shoot architecture variables. The model was calibrated and tested using sufficiently large training and testing data sets, proving that it is robust enough for the prediction of leaf area of the three apple cultivars used in this study, which exhibit a contrasting leaf and shoot morphology. Combined with light response curves measured in 2015 (Bairam et al., unpubl.), the initial architecture thus modeled will help to create a mapping of photosynthetic potential for each leaf. Furthermore, the combination of this information with the developmental kinetics of each fruit on such a branch (Bairam et al., unpubl.) will then allow finding coefficients for daily sugar transport rates from source leaves to fruits.

This study has shown that the distribution of leaves along a shoot of the same type follows certain clear endogenous regularities that seem to be genotype dependent (cf. Lauri, 2007), with rather little phenotypic plasticity. Further experimental and modeling studies will be conducted to try to decipher and quantify the physiological mechanisms behind these regularities, in order to improve the modeling of apple fruit quality in the context of the first-order branch as an experimental system.

## Author contributions

EB, MD, and GBS conceived and designed the work; EB, MD, and CLM collected the experimental data; EB developed the mathematical models, and did the programming in R; EB and GBS analyzed and interpreted the data; EB and GBS wrote the paper; MD revised it critically; all authors approved the final version.

## Funding

The doctoral thesis of EB was funded by a strategic bourse of the Région Pays de la Loire, France, convention no. 2011-12596. All financial help is duly acknowledged.

### Conflict of interest statement

The authors declare that the research was conducted in the absence of any commercial or financial relationships that could be construed as a potential conflict of interest.

## Supplementary material

The leaf data can be accessed here: https://zenodo.org/record/215977. The R scripts used can be found here: https://github.com/EmnaBairam/Apple_leaf_surface

## References

[B1] Anderson J. L. Richardson E. A. (1982). Utilizing meteorological data for modeling crop and weed growth, in Biometeorology in integrated pest management, eds Hatfield J. L. Thomason I. J. (New York, NY: Academic), 449–461.

[B2] Barthélémy D. (1991). Levels of organization and repetition phenomena in seed plants. Acta Biotheor. 39, 309–323. 10.1007/BF00114184

[B3] Björkman O. Demmig-Adams B. (1995). Regulation of photosynthetic light energy capture, conversion, and dissipation in leaves of higher plants, in Ecophysiology of Photosynthesis (Berlin Heidelberg, NY: Springer), 17–47.

[B4] Bos H. J. Neuteboom J. H. (1998). Growth of individual leaves of spring wheat (*Triticum aestivum* L.) as influenced by temperature and light intensity. Ann. Bot. 81, 141–149. 10.1006/anbo.1997.0532

[B5] Buck-Sorlin G. H. (2002). L-system model of the vegetative growth of winter barley, in Fifth *German Workshop on Artificial Life*, eds Polani D. Kim J. Martinetz T. (Lübeck: Akademische Verlagsgesellschaft Aka GmbH), 53–64.

[B6] Buck-Sorlin G. H. (2013). Functional-structural plant modeling, in Encyclopedia of Systems Biology, eds Dubitzky W. Wolkenhauer O. Cho K. Yokota H. (New York, NY: Springer), 778–781. 10.1007/978-1-4419-9863-7

[B7] Buck-Sorlin G. H. Bell A. D. (2000). Models of crown architecture in quercus petraea and q.robur: shoot lengths and bud numbers. Forestry 73, 1–19. 10.1093/forestry/73.1.1

[B8] Casella E. Sinoquet H. (2003). A method for describing the canopy architecture of coppice poplar with allometric relationships. Tree Physiol. 23, 1153–1170. 10.1093/treephys/23.17.1153 14597425

[B9] Chen K. Hu G. Q. Lenz F. (1997). Training and shading effects on vegetative and reproductive growth and fruit quality of apple. Gartenbauwiss 5, 207–213.

[B10] Cohen S. Mosoni P. Meron M. (1995). Canopy clumpiness and radiation penetration in a young hedgerow apple orchard. Agric. For. Meteorol. 76, 185–200. 10.1016/0168-1923(95)02226-N

[B11] Costes E. Crespel L. Denoyes B. Morel P. Demene M. N. Lauri P. E.. (2014). Bud structure, position and fate generate various branching patterns along shoots of closely related Rosaceae species: a review. Front. Plant Sci. 5:666. 10.3389/fpls.2014.00666 25520729 PMC4251308

[B12] Da Silva D. Boudon F. Godin C. Sinoquet H. (2008). Multiscale framework for modeling and analyzing light interception by trees. Multiscale Model. Sim. 7, 910–933. 10.1137/08071394X

[B13] Da Silva D. Han L. Faivre R. Costes E. (2014). Influence of the variation of geometrical and topological traits on light interception efficiency of apple trees: sensitivity analysis and metamodelling for ideotype definition. Ann. Bot. 114, 739–752. 10.1093/aob/mcu034 24723446 PMC4156120

[B14] Dekeyser D. Duga A. T. Verboven P. Hendrickx N. Nuyttens D. (2013). Assessment of orchard sprayers using laboratory experiments and CFD modelling. Biosyst. Eng. 114, 157–169. 10.1016/j.biosystemseng.2012.11.013

[B15] Duga A. T. Ruysen K. Dekeyser D. Nuyttens D. Bylemans D. Nicolai B. M. (2015). Spray deposition profiles in pome fruit trees: effects of sprayer design, training system and tree canopy characteristics. Crop Protec. 67, 200–213. 10.1016/j.cropro.2014.10.016

[B16] Evers J. B. Vos J. Fournier C. Andrieu B. Chelle M. Struik P. C. (2005). Towards a generic architectural model of tillering in Gramineae, as exemplified by spring wheat (*Triticum aestivum*). New Phytol. 166, 801–812. 10.1111/j.1469-8137.2005.01337.x 15869643

[B17] Evers J. B. Vos J. Fournier C. Andrieu B. Chelle M. Struik P. C. (2007). An architectural model of spring wheat: evaluation of the effects of population density and shading on model parameterization and performance. Ecol. Model. 200, 308–320. 10.1016/j.ecolmodel.2006.07.042

[B18] Falster D. S. Westoby M. (2003). Leaf size and angle vary widely across species: what consequences for light interception? New Phytol. 158, 509–525. 10.1046/j.1469-8137.2003.00765.x 36056508

[B19] Fanwoua J. Bairam E. Delaire M. Buck-Sorlin G. (2014). The role of branch architecture in assimilate production and partitioning: the example of apple (*Malus domestica*). Front. Plant Sci. 5:338. 10.3389/fpls.2014.00338 25071813 PMC4089354

[B20] Fisher J. B. (1984). Tree architecture: relationships between structure and function, in Contemporary Problems in Plant Anatomy, eds White R. A. Dickson W. C. (New York, NY: Academic Press), 541–589.

[B21] Fisher J. B. (1986). Branching patterns and angles in trees, in The Economy of Plant Form and Function, ed Givnish T. J. (Cambridge: Cambridge University Press), 493–523.

[B22] Freeman Freeman G. M. Bolas B. D. (1956). A method for the rapid determination of leaf area in the field. Rep. East Malling Res. Stn. 1955, 104–107.

[B23] Gu S. Evers J. B. Zhang L. Mao L. Zhang S. Zhao X.. (2014). Modelling the structural response of cotton plants to mepiquat chloride and population density. Ann. Bot. 114, 877–887. 10.1093/aob/mct309 24489020 PMC4156114

[B24] Han L. Costes E. Boudon F. Cokelaer T. Pradal C. Da Silva D. (2012). Investigating the influence of geometrical traits on light interception efficiency of apple trees: a modelling study with MAppleT, in 2012 IEEE Fourth International Symposium on Plant Growth Modeling, Simulation, Visualization and Applications (PMA), eds Kang M. Z. Dumont Y. Guo Y. (Shanghai: IEEE Press), 152–159.

[B25] Hemmerling R. Kniemeyer O. Lanwert D. Kurth W. Buck-Sorlin G. (2008). The rule-based language XL and the modelling environment GroIMP illustrated with simulated tree competition. Funct. Plant Biol. 35, 739–750. 10.1071/FP08052 32688828

[B26] Hotsonyame G. K. Hunt L. A. (1998). Effects of sowing date, photoperiod and nitrogen on variation in main culm leaf dimensions in field-grown wheat. Can. J. Plant Sci. 78, 35–49. 10.4141/P97-027

[B27] Kahlen K. Stützel H. (2007). Estimation of geometric attributes and masses of individual cucumber organs using three-dimensional digitizing and allometric relationships. J. Am. Soc. Hortic. Sci. 132, 439–446.

[B28] Lauri P. E. (2007). Differentiation and growth traits associated with acrotony in the apple tree (*Malus domestica*, Rosaceae). Am. J. Bot. 94, 1273–1281. 10.3732/ajb.94.8.1273 21636493

[B29] Lauri P. E. Térouanne E. Lespinasse J. M. Regnard J. L. Kelner J. J. (1995). Genotypic differences in the axillary bud growth and fruiting pattern of apple fruiting branches over several years—an approach to regulation of fruit bearing. Sci. Hortic. 64, 265–281. 10.1016/0304-4238(95)00836-5

[B30] Lauri P. É. Trottier C. (2004). Patterns of size and fate relationships of contiguous organs in the apple (*Malus domestica*) crown. New Phytol. 163, 533–546. 10.1111/j.1469-8137.2004.01136.x 33873738

[B31] Lindenmayer A. (1968a). Mathematical models for cellular interactions in development. I. Filaments with one-sided inputs. J. Theor. Biol. 18, 280–299. 10.1016/0022-5193(68)90079-9 5659071

[B32] Lindenmayer A. (1968b). Mathematical models for cellular interactions in development II. *Simple and branching filaments with two-sided inputs*. J. Theor. Biol. 18, 300–315. 10.1016/0022-5193(68)90080-5 5659072

[B33] Massonnet C. Regnard J. L. Lauri P. E. Costes E. Sinoquet H. (2008). Contributions of foliage distribution and leaf functions to light interception, transpiration and photosynthetic capacities in two apple cultivars at branch and tree scales. Tree Physiol. 28, 665–678. 10.1093/treephys/28.5.665 18316299

[B34] Montgomery D. C. Peck E. A. Vining G. G. (2015). Introduction to Linear Regression Analysis. New Jersey, NJ: J. Wiley and Sons.

[B35] Pararajasingham S. Hunt L. A. (1996). Effects of photoperiod on leaf appearance rate and leaf dimensions in winter and spring wheats. Can. J. Plant Sci. 76, 43–50. 10.4141/cjps96-008

[B36] Prusinkiewicz P. (1998). Modeling of spatial structure and development of plants: a review. Sci. Hortic. 74, 113–149.

[B37] Snee R. D. (1977). Validation of regression models: methods and examples. Technometrics 19, 415–428. 10.1016/S0304-4238(98)00084-3

[B38] Spann T. M. Heerema R. J. (2010). A simple method for non-destructive estimation of total shoot leaf area in tree fruit crops. Sci. Hortic. 125, 528–533. 10.1016/j.scienta.2010.04.033

[B39] Štampar F. Hudina M. Usenik V. Šturm K. Marn V. Batič F. (1999). Influence of leaf area on net photosynthesis, yield and flower-bud formation in apple (*Malus domestica* Borkh.). PlantPhysiol. 39, 101–106.

[B40] Sussex I. M. Kerk N. M. (2001). The evolution of plant architecture. Curr. Opin. Plant Biol. 4, 33–37. 10.1016/S1369-5266(00)00132-1 11163165

[B41] Tivet F. Pinheiro B. D. S. Raïssac M. D. Dingkuhn M. (2001). Leaf blade dimensions of rice (*Oryza sativa* L. and *Oryza glaberrima* Steud.). Relationships between tillers and the main stem. Ann. Bot. 88, 507–511. 10.1006/anbo.2001.1447

[B42] Tsukaya H. (2004). Leaf shape: genetic controls and environmental factors. Int. J. Dev. Biol. 49, 547–555. 10.1387/ijdb.041921ht 16096964

[B43] Vos J. Evers J. B. Buck-Sorlin G. H. Andrieu B. Chelle M. de Visser P. H. B. (2010). Functional-structural plant modelling: a new versatile tool in crop science. J. Exp. Bot. 61, 2102–2115. 10.1093/jxb/erp345 19995824

[B44] Zhu Y. Chang L. Tang L. Jiang H. Zhang W. Cao W. (2009). Modelling leaf shape dynamics in rice. NJAS 57, 73–81. 10.1016/j.njas.2009.11.001

